# Niche harmony search algorithm for detecting complex disease associated high-order SNP combinations

**DOI:** 10.1038/s41598-017-11064-9

**Published:** 2017-09-14

**Authors:** Shouheng Tuo, Junying Zhang, Xiguo Yuan, Zongzhen He, Yajun Liu, Zhaowen Liu

**Affiliations:** 1 School of Computer Science and Technology, Xidian University, Xi’an, 710071 P.R. China; 2 School of Mathematics and Computer Science, Shaanxi University of Technology, Hanzhong, 723000 P.R. China

## Abstract

Genome-wide association study is especially challenging in detecting high-order disease-causing models due to model diversity, possible low or even no marginal effect of the model, and extraordinary search and computations. In this paper, we propose a niche harmony search algorithm where joint entropy is utilized as a heuristic factor to guide the search for low or no marginal effect model, and two computationally lightweight scores are selected to evaluate and adapt to diverse of disease models. In order to obtain all possible suspected pathogenic models, niche technique merges with HS, which serves as a taboo region to avoid HS trapping into local search. From the resultant set of candidate SNP-combinations, we use G-test statistic for testing true positives. Experiments were performed on twenty typical simulation datasets in which 12 models are with marginal effect and eight ones are with no marginal effect. Our results indicate that the proposed algorithm has very high detection power for searching suspected disease models in the first stage and it is superior to some typical existing approaches in both detection power and CPU runtime for all these datasets. Application to age-related macular degeneration (AMD) demonstrates our method is promising in detecting high-order disease-causing models.

## Introduction

With the rapid development of high-throughput genotyping technology, single-nucleotide polymorphism (SNP) data increases explosively, which establishes favorable conditions to detect cause of disease for researchers. Though genome wide association study (GWAS) has successfully identified many single SNP genetic variants associated with disease status or phenotypic traits^1–4^ what has been widely acknowledged is that it generally fails to detect high-order SNP-combinations which may be an important contributor to pathogenic factors synergistically affecting disease status^5^. Detecting such model from a dataset with hundreds of thousands of SNPs is facing following two challenges^6^.

The first challenge is the enormous computation burden imposed by the combination explosion of genotype. The number of candidate *k-way* SNP-combinations for a dataset with *n* SNP markers equals $$(\begin{array}{c}n\\ k\end{array})=\frac{n(n-1)\cdots (n-k+1)}{k!}\propto {n}^{k}$$. Obviously, it is unworkable to test all *k-way* SNP combinations at whole-genome scale when *k* > 3, even with high-performance computers available at present. The second challenge arises from the diverse nature of SNP interaction models, such as additive effect model, non-additive effect model and statistical epistasis model. Furthermore, some spurious multi-loci combination models may also be associated with phenotype due to statistics with high degree of freedom, the huge number of hypothesis tested and limited sample sizes^7, 8^ which all could result in a high false discovery rate (FDR).

For the first challenge, several multi-loci detection algorithms^5, 6, 9–22^ have been proposed for improving the detecting speed. SNPHarvester algorithm^9^ uses stochastic strategy to generate multiple paths for identifying *k-way* SNP interaction models. BEAM^23^ introduces a Bayesian partition model and employs a Markov chain Monte Carlo sampling strategy to discover the model with maximum posterior probability. In Boost^5^, Boolean operation is adopted to examine all pairwise SNP interaction using exhaustive search. Sangseob Leem *et al*.^11^ introduces a fast algorithm for detecting high order epistatic interactions by performing clustering with *k*-means algorithm on all SNPs, in which the candidates of *k-way* are selected from the *k* clusters, reducing the number of combinations. Collins RL *et al*. use multifactor dimensionality reduction (MDR) to detect three-locus epistatic interaction^12^, the ReliefF algorithm is used first to select a small candidate set for reducing computation burden. Dynamic clustering and cloud computing^10^ are also employed to detect high-order genome-wide epistatic interaction in which forty virtual machines are constructed for speeding up the detection of multi-locus epistasis. Jonathan *et al*. present a multipoint method for studying the genome-wide association by imputation of genotypes^13^, which is a model-based imputation method for inferring genotypes at observed or unobserved SNPs.

The main problem of these algorithms is their huge computational cost and preference to some types of disease models, e.g., to the models with obvious marginal effect.

Recently swarm based intelligent optimization algorithm attracts much attentions in reducing computational burden due to its power of effectively resolving NP-hard problems in polynomial time. M Aflakparast *et al*. propose Cuckoo search (CS) algorithm^14^ to explore multi-loci epitasis. In the CS, by dividing SNP sites into M groups according to correlation among SNPs, only *k-way* (*k* < = M) SNP combinations are selected out of the M groups. Ant colony optimization (ACO) is adopted in AntEpiSeeker^15^ and MACOED^17^, where the former employs *chi-square test*(*χ* ^2^) score to evaluate association between SNP combinations and phenotype, while the latter adopts Bayesian based K2-score and logistic regression based AIC in screening SNP combinations in the first stage, and *χ* ^2^ is adopted to test the significance difference between control and case in the second stage. Shang J. *et al*. use particle swarm optimization (PSO) to discover SNP-SNP interactions^18^, which uses opposition-based learning, dynamic inertia weight and postprocedure to enhance the search ability of the PSO for finding SNP-SNP interactions. Although swarm intelligent search have the ability to speed up detection process, for high-order disease models unfortunately, they are easy to trap into local search. Table 1 presents the characteristic of the five state-of-the-art algorithms for detecting multi-locus disease-causing models.

**Table 1:** Characteristic of five algorithms (Beam, SNPHarvester, CSE, MACOED, Boost).

Algorithm	Search method	Multi-loci?	Score criteria	drawbacks
BEAM	Markov chain Monte Carlo	Y	Bayesian score B statistic	(1) Having preference to disease models.
SNPHarvester	PathSeeker: heuristic search	Y	Chi-square test	(2) Easily trapping into local search.
CSE	Cuckoo search	Y	Bayesian score	(1) Dividing SNP sites into M groups, which are difficult to determine for unknown datasets.
				(2) Having preference to disease models.
MACOED	Ant colony optimization	Y (only 2-loci)	**1st stage**: Bayesian based K2-score; Logistic regression Based AIC	(1) Logistic regression based AIC suffers from increasing computational complexity for high-order detection.
			**2nd stage**: Chi-square test	(2) Not sui**table** for detecting high-order models.
BOOST	Exhaustive search	Y (only 2-loci)	**1st stage**: Kirkwood Superposition Approximation.	Exhaustive search is not suitable for detecting high-order models.
			**2nd stage**: Chi-square test	Having statistical power limitations

As to the second challenge which relates to diversity of disease models, logistic regression, linear regression, LD- and haplotype-based method, and Bayesian network scoring^23–27^ have been proposed. None of the methods are universally better but with either low statistical power or preference to some types of disease models.

To reduce computation burden and adapt to diversity of various types of disease models, we propose a Niche Harmony Search Algorithm to detect high-order SNP combinations (NHSA-DHSC) associated with complex diseases. It follows two stages: screening and testing. In screening stage, a new niche technique is merged into harmony search algorithm for exploring all suspected disease-causing SNP combinations. To quickly find as more types of disease-causing models as possible, we employ three computationally lightweight and functionally complementary evaluation functions, Bayesian network based K2-score, Gini-score and Joint entropy, for calculating the association between SNP combinations and disease status. The suspected SNP combination models gained from the screening stage are stored into a candidate set (CS). In testing stage, a modified G-test method is used to test the authenticity of the candidate SNP combination models in CS.

Our experiments indicate that the proposed NHSA-DHSC is superior in detection power, running speed and identification ability for diverse disease models compared to current intelligent algorithms.

## Outline

**Figure 1:**
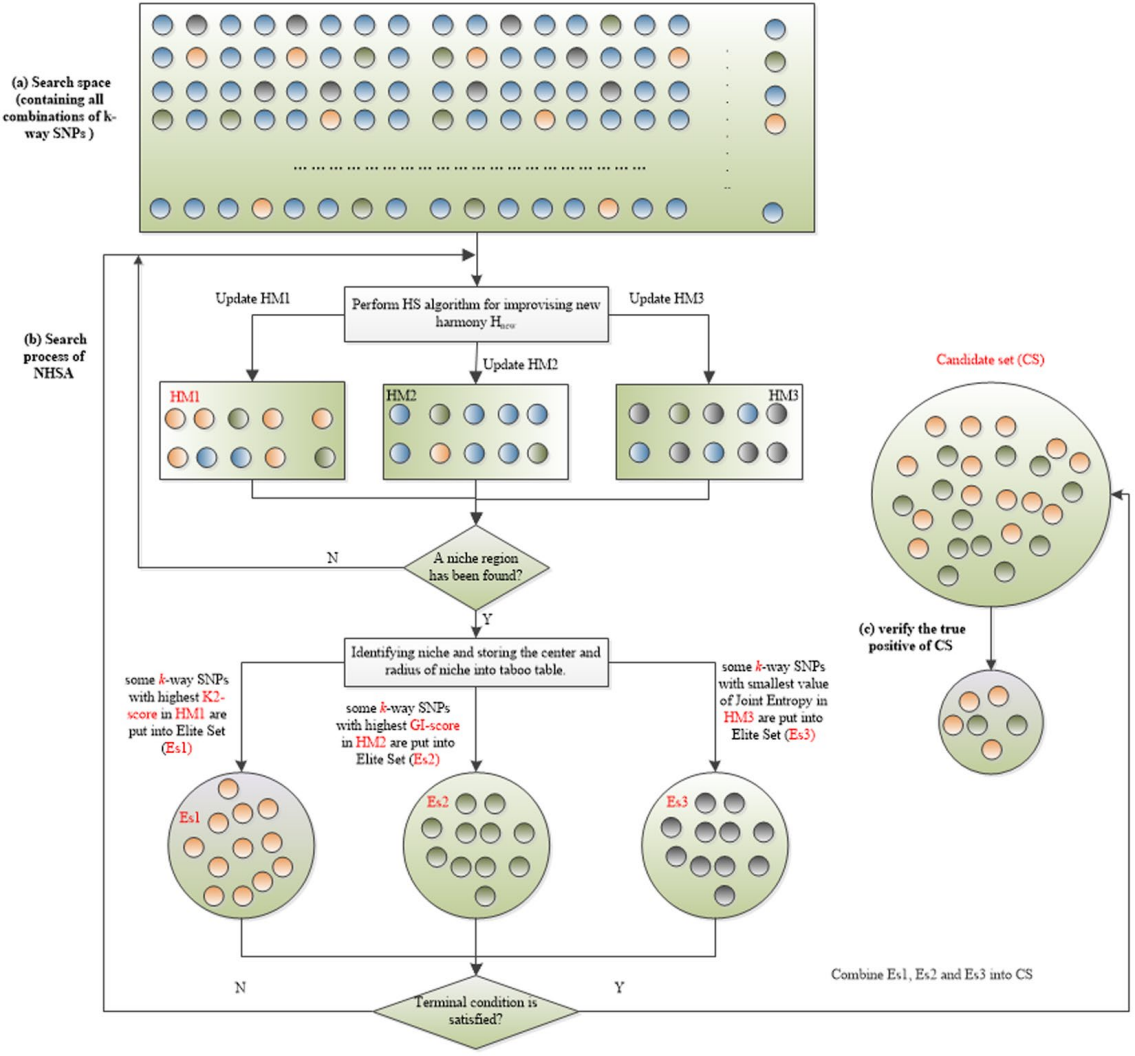
The outline of the NHSA-DHSC algorithm. (**a**) In search space, there are *k n C* balls, where *n* is the number of SNPs in dataset, *k* is the number of SNPs in each *k-way* SNP combination. Each ball in the figure denotes a *k-way* SNP-combination. The NHSA-DHSC aims to find out the true disease-causing *k-way* models from the search space without testing all *k-way* SNP-combinations. (**b**) At first, the HM1, HM2 and HM3 are initialized randomly in search space. Next, in the search process of HS, every newly generated harmony Hnew is used to update the HM1, HM2 and HM3 according to the natural evolutional rules of “Survival of the fittest”. During a period of time, if the HM1, HM2 and HM3 cannot be updated by new generated harmony Hnew, the niche algorithm is automatically triggered to identify new niche region, at the same time, some better solutions are chosen from HM1, HM2 and HM3 separately, so to substitute some worse solutions in the elite sets Es1, Es2 and Es3. Then repeat the search process of HS until the terminal condition is satisfied. After completed the harmony search, the solutions of Es1, Es2 and Es3 are merged into the candidate set (CS). (**c**) In the CS, some solutions are no significantly associated with the phenotype. G-test statistic is adopted to verify the true positive of each SNP-combination.

Figure 1 presents the outline of the NHSA-DHSC algorithm. The goal of the first stage is to quickly find all suspected *k-way* disease-causing models from all *k-way* SNP combinations where the *k-way* disease-causing model denotes a k-SNPs combination that has joint effect on the disease status (k is the number of SNPs). It is responsible for significantly increasing the risks of complex diseases^28, 29^.

As shown in Fig. 1, there are ${C}_{n}^{k}$ *k-way* SNP combinations, in which only a few models are disease-causing ones. Niche technology is used to obtain as many types of pathogenic models as possible from a large number of SNP combinations. To search for them efficiently, harmony search (HS) algorithm is used and three harmony memories HM1, HM2 and HM3 are employed to store candidate solutions: HM1 stores candidate SNP combinations screened by Bayesian network based K2-score, and HM2 stores the ones by Gini-score. The two scores are complementary in that the K2-score is superior in identifying models with low marginal effects and gini-score is, compared to K2-score, more capable of identifying high-order models when their genetic heritability (H^2^) is very low^30^. Joint entropy is adopted to evaluate the harmonies in HM3. Unlike K2-score and Gini-score, joint entropy is a heuristic factor for guiding the HS algorithm to quickly explore the high-order disease models with *very low or even no* marginal effects (DNME). We propose joint entropy as a heuristic factor since we found via our data experiments that the factor is really powerful in identifying some high-order disease models with even no marginal effect, for which the K2-score and Gini-score are powerless.

Our screening process is as follows.

- Initialize HM1, HM2 and HM3 by selecting *k-way* SNP-combinations randomly from all *k-way* SNP-combinations.
- Generate new solution H_new_ repeatedly using HS rules to update the HM1, HM2 and HM3 according to the natural evolutional theory of “Survival of the fittest” (see algorithm (1) in Methods section).
- Until when HM1, HM2 and HM3 cannot be further updated, niche algorithm is automatically triggered to identify new niche (see algorithm (2) in Methods section). Meanwhile, some best solutions are chosen from HM1, HM2 and HM3 separately to substitute some worse solutions in elite sets Es1, Es2 and Es3.
- Reinitialize the HM1, HM2, and HM3 randomly, where the solutions from identified niche regions cannot be visited and evaluated for avoiding repeated search in the niche regions.
- When terminal condition is satisfied, solutions in Es1, Es2 and Es3 are merged and stored in candidate set (CS).

(Testing Stage) Some spurious disease models may be included in the CS in the first stage. In the second stage, we adopt modified G-test method^31^ to further verify the authenticity of the candidate models in CS.

In this study, the niche technique is mainly to discover some SNP-combinations with strong marginal effect and make the HS find all suspected disease-causing models, where the marginal effects do not only come from single SNP markers, but also may be synergistic effects of multi-SNP makers. In the search process of HS, the position and size of each niche region are recorded into a taboo table for forcing the HS algorithm to search new solutions in unexplored regions. In this way, all possible *k-way* SNP combination models having strong association with phenotype can be extracted one by one. The detailed process of the NHSA-DHSC algorithm is introduced in Methods section and the related techniques are presented in supplementary info file.

## Experimental Results

20 simulated disease models, which contain twelve 2-locus Disease models with Marginal Effects (DME) and eight high-order Disease models with No Marginal Effect (DNME), are employed to investigate the performance of the NHSA-DHSC algorithm. The simulation datasets for the 20 disease models, real Age-related macular degeneration (AMD) data, and seven evaluation metrics are introduced in Methods section in detail. We compare the experimental results of NHSA-DHSC with those gained from five state-of-the-art algorithms (BEAM^23^, SNPHarvester^9^, BOOST^5^, CSE^14^ and MACOED^17^).

### Experimental results for simulation datasets

#### Detecting DME disease models

**Figure 2:**
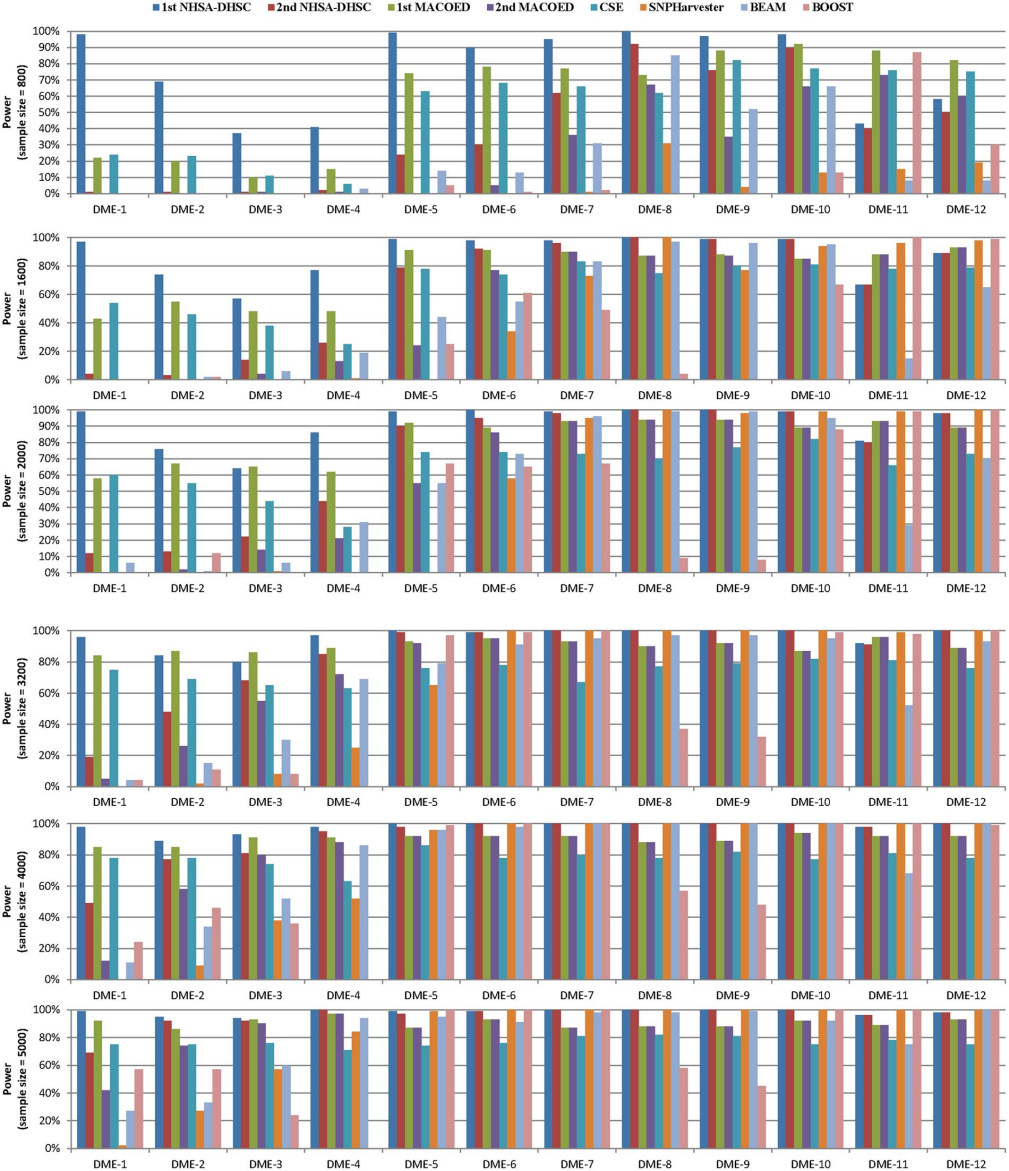
Power comparison on 12 DME models with 100SNPs: In each bar chart, there are eight power bars. The NHSA-DHSC and MACOED have two powers (1st power and 2nd power), where the 1st power denotes the detection power in the first search stage, in other word, it is the ability to obtain the disease-causing models and put them into the candidate set (CS) in the first stage; the 2nd power denotes the rate that the disease-causing model can survive into the final results. For other algorithms, the power denotes the rate that the disease-causing model can be found and determined. In Fig. 2, six bar charts display the detection power of six algorithms on 12 DME models with different sample sizes (800, 1600, 2000, 3200, 4000, 5000).

**Figure 3:**
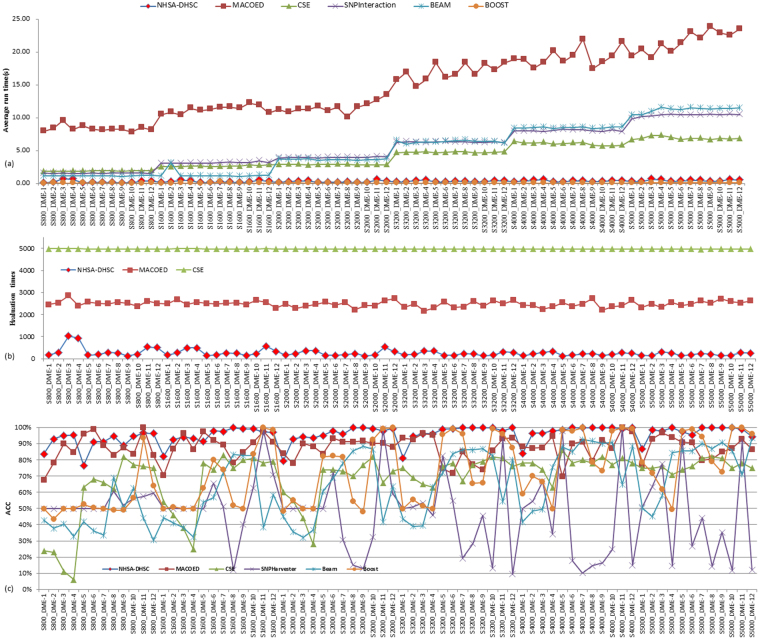
Comparison of six algorithms on Runtime, ACC and the average number of Evaluating SNP combinations for finding the disease-causing models from dataset with 100SNPs. The labels on horizontal axis denote disease models, where Sn-M denotes dataset containing disease model M includes n samples. For example, S800-DME-1 denotes the DME-1 model with 800 samples. Figure 3(a) display the average Runtime for finding the disease-causing models. Figure 3(b) compares the number of evaluating SNP combinations using three intelligent optimization algorithms to find the disease-causing models, which aims to compare the capability of three intelligent algorithms (NHSA-DHSC, MACOED and CSE) on reducing the computational burden. Figure 3(c) presents the ACC value of algorithms for solving 12 * 6 = 72 DME models.

We first investigate the proposed algorithm on 12 DME data sets with 100 SNP markers, which aims to test the sensitive of our method for dataset with different sample size. The bar charts shown in Fig. 2 present the power of six algorithms to solve 12 DME models with sample size 800, 1600, 2000, 3200, 4000 and 5000. Figure 3 displays the runtime, mean evaluation times (MEs) and ACC value of all methods. Other three statistical metrics TPR, SPC and FDR are summarized in Table E-3 (see supplementary info file), and the TPR and SPC are presented using point line chart in Fig. E-4 (see supplementary info file).

(**Analysis of Detection Power**) As can be observed from Fig. 2, for most of DME models, the detection power of NHSA-DHSC in the first search stage outperforms the other five methods. Especially, for DME-1~DME-4, NHSA-DHSC has very obvious advantages. In the second stage, the power of NHSA-DHSC decreases apparently on DME-1~DME-4 because some disease-causing models fail to pass threshold p-value from G-test statistics (Bonferroni correction), which makes the true positive rate (TPR) decrease. The MACOED is similar to the NHSA-DHSC algorithm; it employs the ACO algorithm to search the candidate solutions in the first phase and uses the chi-square to further test the authenticity in the second phase. Almost on all DME models, the power of our approach is higher than that of MACOED in two corresponding stages. The NHSA-DHSC also has obvious advantage on power over BEAM, SNPHarvester and Boost, and the 1^st^ power of it obviously outperforms the CSE.

(**Ability for reducing the computational burden**) Fig. 3(a) indicates that the Boost takes the least Runtime among six methods, but the NHSA-DHSC takes less Runtime than MACOED, CSE, BEAM and SNPHarvester apparently, and the Runtime of NHSA-DHSC increases very slow with the increasing sample size but MACOED, CSE, BEAM and SNPHarvester are converse. In Fig. 3(b), NHSA-DHSC uses a very small number of evaluations to detect pathogenic models, and significantly lower than MACOED and CSE, which demonstrates that our approach reduces the computational burden effectively.

(**Performance on TPR, FDR, SPC and ACC**) As shown in Fig. 3(c), for ACC, our method outperforms other five algorithms on the majority of models. It is indicated in Table E-3 that, for DME-2~DME-4, all of algorithms has poor performance on TPR and FDR when the sample size is less than 2000, where the TPR of MACOED is higher than that of NHSA-DHSC, but the FDR of NHSA-DHSC is very lower than that of MACOED, which demonstrates that the G-test in our method is more stringent for avoiding false positive rate than chi-square of MACOED. We can find from Fig. E-4 (see supplementary info file) that the NHSA-DHSC and Boost have highest SPC value among six algorithms but the NHSA-DHSC is superior to Boost on TPR apparently. And NHSA-DHSC is very outstanding for all datasets with different sample size except for DME-2~DME-4 with small sample size (< = 2000) (see Fig. E-5 Supplementary info file).

In supplementary info file, we also present the experiment results and analysis for dataset with 1000 SNPs.

To sum up, for DME models, our method decreases the computational burden effectively and its detection power, SPC, ACC and FDR are superior to most of compared algorithm, which demonstrates the NHSA-DHSC is promising to detect DME models.

#### Detecting DNME disease models

For 8 high-order DNME models, we compare NHSA-DHSC with three state-of-the-art heuristic search algorithms which can detect the high-order SNP combination associating with complex diseases. The detailed experimental results are summarized in Table 2.

**Table 2:** Eight high-order DNME models.

Algorithm	metrics	DNME-1	DNME-2	DNME-3	DNME-4	DNME-5	DNME-6	DNME-7	DNME-8
**NHSA-DHSC**	1^st^ Power	100%	100%	100%	100%	100%	100%	2%	100%
	2^nd^ Power	100%	100%	100%	100%	100%	100%	2%	100%
	TPR	100%	100%	100%	100%	100%	100%	2%	100%
	SPC	100%	100%	100%	90%	100%	100%	98%	100%
	ACC	100%	100%	100%	95%	100%	100%	50%	100%
	FDR	0%	0%	0%	9%	0%	0%	50%	0%
	MEs	1093	1081	2342	4415	2175	3870	50001	3849
	Runtime	1.48	1.44	4.1	7.67	3.87	7.61	102.41	9.38
**BEAM**	Runtime	5.75	5.62	5.88	5.7	5.76	5.74	5.75	5.73
	Power	38%	6%	0%	0%	0%	0%	0%	0%
	TPR	38%	6%	0%	0%	0%	0%	0%	0%
	SPC	100%	100%	100%	100%	100%	100%	100%	100%
	PPV	100%	100%	100%	100%	100%	100%	100%	100%
	ACC	59%	46%	30%	44%	43%	44%	45%	43%
	FDR	0%	0%	0%	0%	0%	0%	0%	0%
**SNPHarvester**	Power	0%	0%	0%	0%	0%	0%	0%	0%
	Runtime	7.64	7.55	12.94	13.62	13.47	17.67	17.91	17.47
	TPR	0%	0%	0%	0%	0%	0%	0%	0%
	SPC	100%	100%	98%	100%	85%	100%	100%	100%
	ACC	50%	50%	50%	50%	46%	50%	50%	50%
	FDR	0%	0%	100%	0%	100%	0%	0%	0%
**CSE**	Power	53%	52%	9%	8%	8%	3%	1%	1%
	MEs	49856	49855	49795	49788	49791	49712	49718	49709
	Runtime	71.89	66.63	110.92	110.06	110.51	252	238.62	275.23
	TPR	53%	52%	9%	8%	8%	3%	1%	1%
	SPC	53%	52%	9%	8%	8%	3%	1%	1%
	ACC	53%	52%	9%	8%	8%	3%	1%	1%
	FDR	47%	48%	91%	92%	92%	97%	99%	99%

Seen from Table 2 is that the NHSA-DHSC is obviously superior to other algorithms in terms of power and Runtime except for DNME-7, and it finds the high-order disease-causing models successfully using very few number of evaluating SNP combination models. For DNME-1 with 100 SNP sites, there are 161700 *3-way* combination models (${C}_{100}^{3}=161700$), the NHSA-DHSC algorithm can identify out the disease-causing model by evaluating 1093 combination models from all *3-way* combinations. For DNME-8, the disease-causing models can be identified from 75287520 *5-way* SNP combinations by evaluating 3849 *5-way* models. Therefore, we believe that the NHSA-DHSC algorithm is promising in detecting high-order SNP combinations associated with complex diseases.

### Experiments on AMD data

#### Detection on all 103611 SNP loci of AMD dataset

NHSA-DHSC algorithm are employed to detect *k-way* (k = 2, …, 4) SNP combinations associated with the AMD. The corresponding results are respectively listed in sheet S-2~S-4 of Supplementary Dataset File 1.

**Figure 4:**
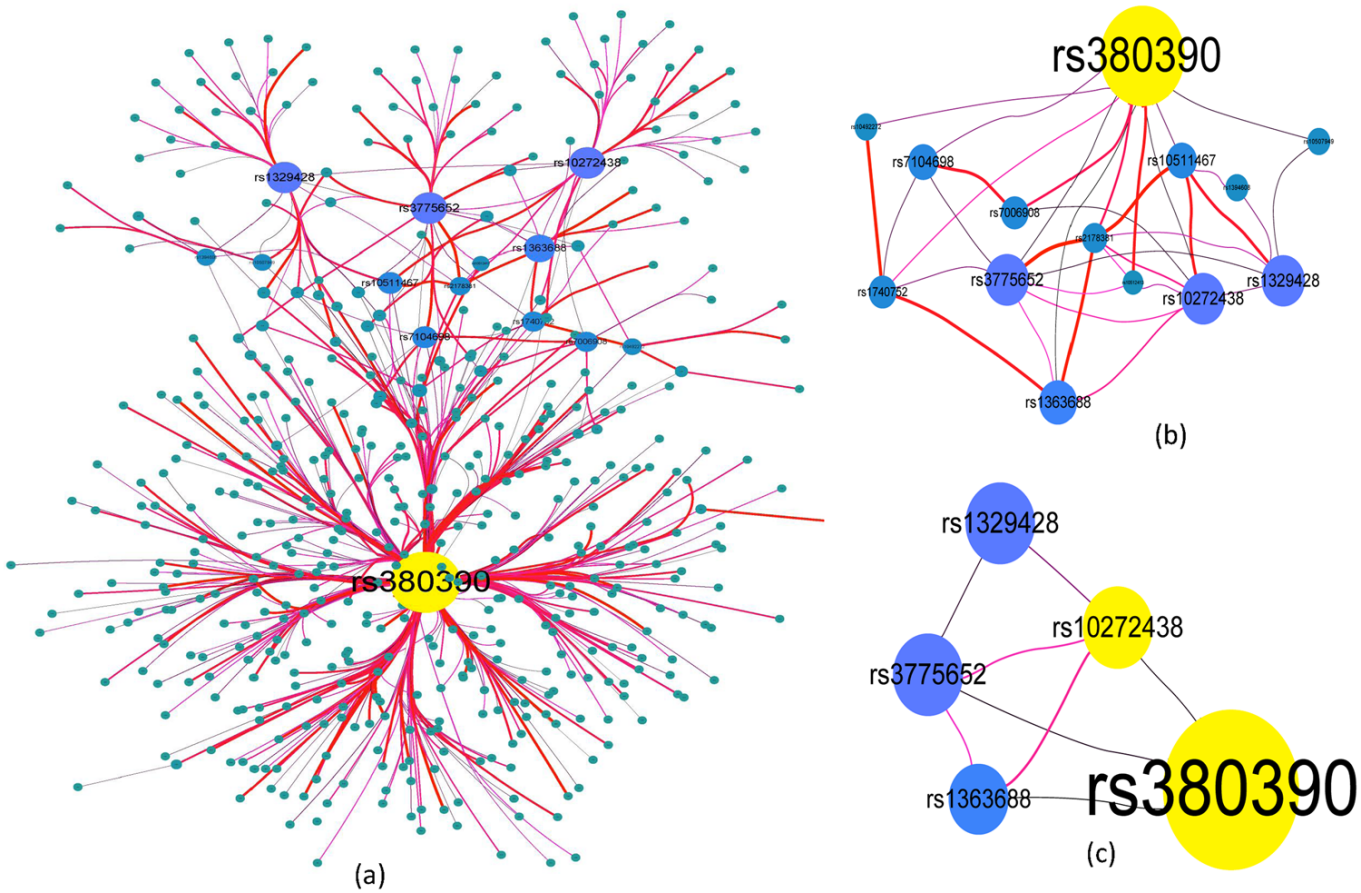
2-way SNP interaction network. (**a**) There are 568 edges and 571 nodes in Fig. 4(a), where each node denotes a SNP locus, the larger size the node, the more number of nodes associated with it. An Edge represents a SNP combination that has strong association with the phenotype, the thicker the edge, the stronger association with the phenotype the SNP combination has (the less p-value from G-test). (**b**) The nodes are selected from Fig. 4 (a). There are 14 nodes and 36 edges in this network. Each node has more than 5 adjacent edges in Fig. 4(a). (**c**) The nodes are also chosen from Fig. 4(a). There are 5 nodes and 8 edges in this network. Each node has more than 10 adjacent edges in Fig. 4(a).

In Fig. 4(a), 2*-way* SNP combination network is created using software Cytoscape 3.3^32^ (http://www.cytoscape.org/). There are 571 nodes and 565 edges in the network. The edge of the network denotes a *2-way* SNP-combination with p-values from G-test (case and control) less than 1e-8. The Node in the network represents a SNP locus which has joint effect with adjacent nodes on phenotype. Figure 4(b) and (c) are the sub-networks of Fig. 4(a). The SNP nodes of Fig. 4(b) have more than 5 adjacent nodes in the network of Fig. 4(a), and the nodes of Fig. 4(c) have more than 10 adjacent nodes in the network of Fig. 4(a).

**Figure 5:**
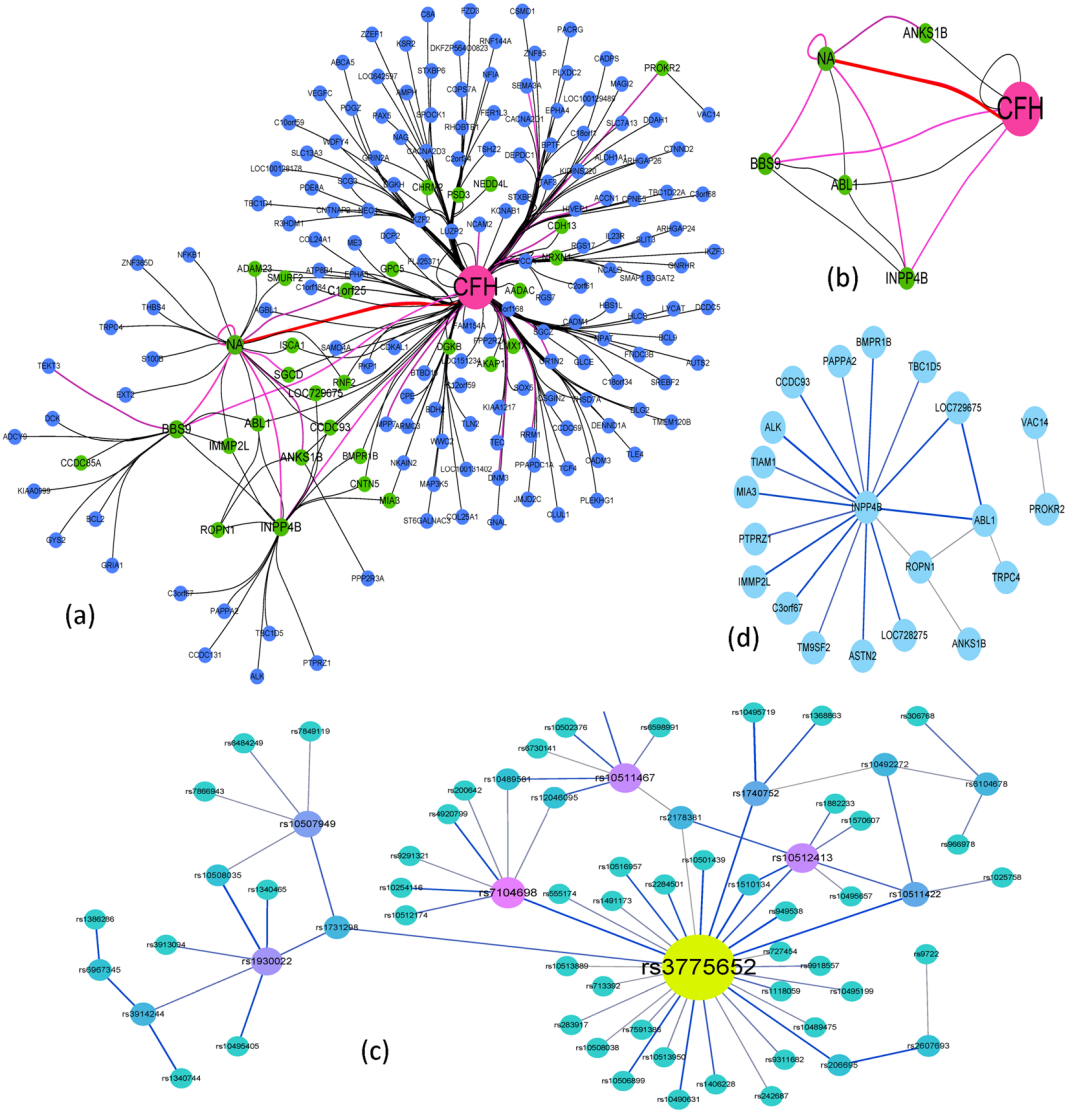
Gene interaction network. Each node denotes a gene locus, the larger size the node, the more number of nodes associated with it. An Edge represents a gene-gene interaction. The thicker the edge, the more number of SNP-pairs linked the two genes. Figure 5(a) is a gene network in which each gene is mapped from SNPs in Fig. 4(a). (**b**) is a sub-network of (**a**), where each gene node has more than 5 adjacent edges in Fig. 5(a). Figure 5(c) is the SNP association network for AMD data filtered five important SNPs (‘rs380390’, ‘rs10272438’, ‘rs1329428’, ‘rs1363688’, ‘rs1394608’). There are 78 edges and 75 nodes in this network. Figure 5(d) is the gene interaction network mapped from SNP network Fig. 5(c).

Figure 5(a) is the gene interaction network that is mapped from SNP network in Fig. 4(a), in which each edge connecting two genes denotes two SNP loci in the two genes are associated with phenotype. Figure 5(b) presents the interaction relationship of the six important genes (*CFH*, *BBS9*, *NA*, *INPP4B*, *ABL1* and *ANKS1B*). In Fig. 5, the thicker the adjacent edge, the more number of SNP-pairs linked the two genes. In Fig. 5(a), there are 260 adjacent edges between CFH and NA.

#### Detection of the remaining SNP loci of AMD dataset (removed five important SNPs)

In order to further investigate the unknown disease models from AMD dataset, we first remove five widely reported SNPs (‘*rs380390*’,‘*rs10272438*’,‘*rs1329428*’,‘*rs1363688*’,‘*rs1394608*’) from AMD data. Then the proposed NHSA-DHSC is used to detect high-order SNP-combinations associated with AMD from the remaining SNP loci. The results are listed in sheet 2 of Supplementary Dataset File 2.

Figure 5(c) shows the 2-way SNP interaction network in which there are 78 edges, where each edge denotes a SNP-pair associating with disease status (the *p-value* cutoff is 1e-8). Figure 5(d) is the gene interaction network mapped from SNP network Fig. 5(c).

#### Experimental results analyses

As shown in Fig. 4, many SNP nodes are connected with three important nodes *rs380390*, *rs1329428* and *rs10272438*. Degrees of the three SNP nodes are respectively equal to 421, 32 and 34, where the *rs380390* and *rs1329428* (both in an intron of the *CFH* gene) have been widely believed to be associated with the AMD^10, 18, 30, 33–38^, the *rs10272438* (in the *BBS9* gene) also has been reported in **[**36, 37, 43, and 44**]**. In addition, the *rs1363688* (degree = 12) and *rs7104698* (degree = 9), which are not in known gene regions, have been separately reported in refs 10, 18, 30 and refs 30 and 39. To our best knowledge, the *rs3775652* (degree = 36 in the network of Fig. 4(a)) has not been reported by other researchers, it is in the gene *INPP4B* that is an oncogenic regulator in human colon cancer^40^. Another SNP *rs1394608* in the *SGCD* gene has also been reported recently in refs 30, 39, 41–43. It indicates in Fig. 4(c) that there is no edge between the *rs380390* and *rs1329428*, the G-test p-value of SNP pair (*rs380390*, *rs1329428*) is equal to 3.24 × 10^−6^ that is larger than the threshold p-value 1.0 × 10^−8^. However the *rs10272438* and *rs3775652* are connected with all other SNP nodes. As a consequence, we speculate that *rs380390* and *rs1329428* may be the disease-causing variation locus and both they were independently associated with Age-related macular degeneration (AMD), but there is very low synergetic effect to AMD among them. The two SNPs may be the potential driver variation locus to AMD and the *CFH* is the potential driver gene on the basis of single nucleotide variations.

In the gene network of Fig. 5, there are a total of 188 gene nodes, where six nodes (*CFH*, *BBS9*, *NA*, *INPP4B*, *ABL1* and *ANKS1B*) have the most adjacency edges. There are 177 SNPs mapped to the *CFH* gene, 16 SNPs mapped to the *BBS9* gene, 17 SNPs mapped to the *INPP4B* gene, 5 SNPs mapped to the *ABL1* gene and 5 SNPs mapped to the *ANKS1B* gene. The NA (375 SNPs are mapped to NA) denotes non-gene coding region. In these genes, the gene *CFH* has been widely believed to be associated with age-related macular degeneration (AMD) disease^44^. Although the other five genes have not been reported to be related to the AMD, they are all associated with other complex disease. For example, it has reported that the gene *BBS9* is associated with consanguineous Pakistani family with Bardet Biedl syndrome^45^, *INPP4B* is an oncogenic regulator in human colon cancer^40^ and is upregulated and functions as an oncogenic driver through SGK3 in a subset of melanomas^46^. Other four important genes (*MPP7*﻿^47^,*ABL1* ^48^, *ANKS1B* ^49, 50^, and *IMMP2L* ^51, 52^) are also associated with somatic mutations in cancers.

As shown in Fig. 5(c), there are five SNPs (‘*rs3775652*’, ‘*rs7104698*’, ‘*rs10511467*’, ‘*rs10512413*’, ‘*rs1930022*’) having more adjacent nodes, where the SNP ‘*rs3775652*’ is in gene I*NPP4B*, and SNP *rs10512413* is in gene *ABL1*, other three SNPs are in non-gene coding region (NA). We can see evidently from Fig. 5(d) that the gene *INNP4B* is related with many genes. Therefore, it can be speculated the SNPs ‘*rs3775652*’ and gene *INPP4B* should also be important effect on AMD.

Tables 3 and 4 list top eight *3-wa*y SNP-combinations with p-values from G-test less than 1e-11 and top eight *4-way* SNP-combinations with p-values from G-test less than 1e-12, respectively.

**Table 3:** Top eight 3-way SNP-combinations with p-values from G-test less than 1e-11 (the last three columns respectively are the p-value of G-test, p-value of chi-square and the prediction accuracy of SVM (Support Vector Machine)).

SNP1			SNP2			SNP3			3-way SNP combination		
name	chromo	p-value	name	chromo	p-value	name	chrom	p-value	G-test	Chi-sq	SVM
***rs380390***	1	6.2E-07	*rs2421596*	2	9.8E-02	*rs555174*	21	7.9E-04	1.2E-12	0	82.2%
*rs3915771*	5	7.7E-04	*rs1360333*	1	9.1E-02	***rs380390***	1	6.2E-07	3.0E-12	0	74.7%
*rs3915771*	5	7.7E-04	***rs380390***	1	6.2E-07	*rs2377257*	3	8.8E-01	5.7E-12	0	76.7%
*rs10515262*	5	1.9E-03	*rs10501439*	11	1.6E-04	***rs1363688***	5	3.8E-05	7.6E-12	0	77.4%
*rs1360333*	1	9.1E-02	***rs380390***	1	6.2E-07	*rs1943581*	18	2.8E-01	8.0E-12	0	74.7%
*rs417637*	17	2.2E-03	***rs10272438***	7	9.7E-06	***rs1363688***	5	3.8E-05	8.3E-12	0	76.7%
***rs10272438***	7	9.7E-06	*rs10512413*	9	2.1E-04	*rs1510134*	4	7.8E-04	8.6E-12	0	80.1%
***rs380390***	1	6.2E-07	*rs1943581*	18	2.8E-01	*rs2377257*	3	8.8E-01	9.7E-12	0	70.5%

**Table 4:** Top eight 4-way SNP-combinations with p-values from G-test less than 1e-12 (the last three columns respectively are the p-value of G-test, p-value of chi-square and the prediction accuracy of SVM (Support Vector Machine)).

SNP1			SNP2			SNP3			SNP4			4-way SNP combination		
name	chrom	p-value	name	chrom	p-value	name	chrom	p-value	name	chrom	p-value	G-test	Chi-sq	SVM
**rs2157998**	7	8.5E-01	**rs1334722**	10	5.7E-02	**rs1740752**	10	4.0E-05	**rs7104698**	11	1.6E-04	2.0E-13	0	80.8%
**rs10508291**	10	1.7E-03	**rs417637**	17	2.2E-03	***rs10272438***	7	9.7E-06	**rs10518080**	4	2.5E-02	6.5E-13	0	80.1%
rs10489581	1	2.1E-03	rs10517007	4	8.0E-01	rs10511467	9	2.9E-05	rs10507949	13	6.8E-05	6.8E-13	0	80.8%
rs961360	2	1.0E-03	***rs380390***	1	6.2E-07	rs10516957	4	5.1E-03	rs10501442	11	9.1E-03	6.8E-13	0	79.5%
***rs3775652***	4	3.7E-07	rs1740752	10	4.0E-05	rs7104698	11	1.6E-04	rs10514569	16	5.7E-02	7.6E-13	0	77.4%
***rs10272438***	7	9.7E-06	***rs380390***	1	6.2E-07	rs10501267	11	7.1E-01	rs920799	18	4.5E-02	9.5E-13	0	81.5%
rs10489581	1	2.1E-03	rs10511467	9	2.9E-05	rs10507949	13	6.8E-05	rs1025758	4	4.0E-03	9.6E-13	0	76.0%
***rs10272438***	7	9.7E-06	rs10482918	21	4.5E-02	rs200642	20	3.7E-04	rs6104678	20	2.1E-04	1.0E-12	0	78.1%

We can see from Table 3 that the eight *3-way* SNP-combinations all contain the SNP locus that have strong marginal effect to AMD, such as *rs380390*, *rs10272438* and *rs1363688*.

In Table 4, there are three 4-way SNP-combinations (*rs2157998*, *rs1334722*, *rs1740752*, *rs7104698*), (*rs10489581*, *rs10517007*, *rs10511467*, *rs10507949*), (*rs10489581*, *rs10511467*, *rs10507949*, *rs1025758*) that don’t contain the known SNP locus associating with AMD and each SNP locus has low marginal effect. We can see from the last column that the SVM prediction accuracies of these SNP combinations are all larger than 75%, it may be worth to study for biologist.

In addition, we can notice that the all p-values from chi-square equal zero in Tables 3 and 4, which demonstrate that the chi-square loses efficacy for testing the association of high-order SNP-combinations when the number of samples is not big enough.

## Discussion

In this work, we propose NHSA-DHSC algorithm to detect high-order SNP combinations associating with the phenotype. And the experimental results demonstrate it has strong global exploration power for detection of high-order disease-causing models from thousands of SNPs. Compared to the existing algorithms, it has following advantages.

1. For high-order disease-causing models in which some individual SNP locus have strong marginal effects, existing intelligent optimization algorithms are easily trapped into local search, resulting in repeated search in a small region (part of SNPs), leading to the loss of the optimal solution. To tackle the problem, our method adopts niche technique to dynamically identify the SNP locus with marginal effect and then uses taboo table to store the identified SNP locus, which can effectively avoid repeated search in a local region and find all possible disease-causing models in a short time.
2. For diverse disease-causing models, to our best knowledge, many existing algorithms usually employ single scoring method (e.g. statistical test method, Bayesian network, and regression method) to identify disease models, which usually results in preference to some specific types of disease model and failure to identify other types of disease models. To address the problem, NHSA-DHSC employs two lightweight identification methods (Bayesian network based K2-score and Gini-score), which has been turned out to be complementary each other in literature^30^.
3. For some high-order disease-causing models with very low or even no marginal effect, existing intelligent optimization algorithms (e.g. MACOED, CSE) and heuristic algorithms (BEAM) always are powerless, which is because no suitable heuristic factor can be found by traditional scoring methods for detecting the disease models. After a large amount of experiments, we find that joint entropy can provide some heuristic clues for guiding the HS to search the high-order disease models. Therefore, in this work, we employ three lightweight and complementary evaluation methods to identify the disease-causing models, where the joint entropy is utilized as heuristic factor to explore the disease models with very low or even no marginal effects.
4. The experimental results on simulation DME datasets demonstrate that the proposed NHSA-DHSC algorithm has very high detection power in the first stage and very low FDR values. Compared with two outstanding intelligent search algorithms MACOED and CSE, our method has very obvious advantages on runtime, power, MEs and FDR.
5. For eight high-order DNME models, our method can quickly find the disease-causing models except for DNME-7, which demonstrates that our method has ability to detect some of high-order disease models.
6. In the real AMD experiment, the NHSA-DHSC successfully found some widely reported SNP locus (e.g. ‘*rs380390*’, ‘*rs1329428*’, ‘*rs10272438*’, ‘*rs1363688*’) associated with AMD and also found some new SNP markers (e.g. ‘*rs3775652*’) that are associated with AMD, we notice that two SNPs (‘*rs380390*’, ‘*rs1329428*’) widely believed to be associated with AMD may be different driver factors to AMD, they have not strong synergistic effect to AMD. In the analysis of gene network, many SNP-combinations are mapped into genes *CFH*, *BBS9*, *ABL1*, *ANKS1B*, *IMMP2L*, *INPP4B*, *SGCD* and non-coding regions, where *CFH* has believed to be associated with AMD, the other genes also have associated with some complex diseases. Therefore, we can speculate that the SNP (‘*rs380390*’,‘*rs10272438*’) and gene *CFH* are associated directly with AMD; others may be indirectly associated with AMD. In other words, SNP (‘*rs380390*’,‘*rs10272438*’) and gene *CFH* may be driver loci to AMD, others are passengers.

However, the NHSA-DHSC is not a flawless method, it also has some shortcomings:

1. For multiplicative models with small size of sample, it has low performance on TPR because some true disease-causing models cannot pass to the test of G-test, which demonstrates the G-test is also not good enough to adapt all disease-models.
2. For some high-order DNME models, it is also powerless, such as DNME-7.

## Methods

In this section, we first define a mathematical model for detecting *k-way* SNP combination in section 5.1. In section 5.2, the proposed NHSA-DHSC algorithm is introduced in detail, which involves the niche identification algorithm. In section 5.3, we introduce three scoring functions for calculating the association between *k-way* SNP combination and disease status and analyze the simulation datasets in section 5.4. In section 5.5, we present seven evaluation metrics for comparing the performance of six algorithms. The parameters setting for six algorithms are introduced in section 5.6.

### Mathematical model for detecting *k-way* SNP combination

Let a set of SNP variables {*X* = *X*_1_, *X*_2_,…, *X*_N_} indicate *N* SNP markers for *n* individuals (samples), *Y* be the phenotype variable with values of {*y*1, *y*_2_,…, *y*_J_}; we represent the homozygous major allele, heterozygous allele and homozygous minor allele as 0, 1 and 2, respectively. For a *k-way* combination model, *I* denotes the number of genotype combinations (there are 3^*k*^ genotype combinations for a *k-way* SNP variables), *J* is the number of phenotype states *Y* (which is equal to 2 for a case-control dataset). *n*_*i*_ is the number of samples in the dataset with SNP loci taking the value of i^th^ genotype combination, *n*_*ij*_ represents the number of samples that the i^th^ genotype combination actually associated with phenotype *y*_*j*_.

**Definition (high-order association**). Let *S* = {*S*_1_, *S*_2_,…*S*_*K*_}(1 < *N*, *s*_*i*_ ∈ *X*) be a set with k SNP loci. *ϕ*(*S*, *Y*) is a score function for evaluating the association between *S* and phenotype *Y*. The *k-way* SNP combination *S* is said to be synergistically associated with phenotype *Y* if and only if $\forall {S}^{\text{'}}\subset S\wedge \varphi (S,Y)\succ \varphi ({S}^{\text{'}},Y)$($\succ$ is a binocular operator for comparing the association strength), and it is said to be associated strongly with $Y$ if $\varphi (S,Y) > \theta$ (is threshold value).

The optimization problem for finding a *k-way* disease-causing combination model can be expressed as

$$\mathop{\max }\limits_{X}\,f(X,Y),X=({X}_{{S}_{1}},{X}_{{S}_{2}},\cdots {X}_{{S}_{k}})$$

where, ${s}_{i}(i=1,2,\mathrm{...},k)$ is the location of i^th^ SNP site, ${X}_{{s}_{i}}$ denotes the value of the ${s}_{i}-th$ SNP marker, ${X}_{{s}_{i}}\ne {X}_{{s}_{j}}(i\ne j)$.$f(X,Y)$ denotes the objective function for evaluating the association between genotype $X$ and phenotype $Y$.

### Niche Harmony Search Algorithm

Harmony search (HS) algorithm is a swarm intelligent optimization algorithm^53^. It mimics the process of improvising a musical harmony when a music orchestra is aiming at composing the most harmonious melody (see standard HS algorithm in supplementary info file). HS algorithm does not dependent on substantial gradient information and an initial search point and it can solve both continuous and discrete combination optimization problems, efficiently. However, it is still not good enough to solve complex multimodal optimization problems if our demand for multiple candidate solutions is more prominent. Recently, some strategies are adopted to find multiple solutions in the study of intelligent optimization algorithm. For example, in refs 14,15,17 and 18 elite set are employed to store optimal and suboptimal solutions, but the solutions in elite set are likely only from one local region, which might make other solutions be lost if the search algorithm has been trapped into a local search. In recent years, niche techniques have received extensive attention for obtaining all possible candidate solutions, which can effectively enhance the search capability of HS owing to avoiding repeatedly search in a small region.

For an optimization task, each harmony corresponds to be a vector consisting of *k* decision variables. Some good harmonies form a harmony memory (HM) which later would be used for creating better harmonies. The harmony memory size (HMS) is defined as the number of harmonies in HM.

In this study, each harmony of HM denotes a *k-way* SNP combination, and the goal of optimization is to find some best harmonies (*k-way* model) which are associating with disease status *Y*.

To explore as many suspected *k-way* genetic variations as possible, we propose a niche HS algorithm (named NHSA-DHSC) for detecting *k-way* SNP combinations associated with phenotype, in which niche strategy^54, 55^ are merged into HS algorithm for enhancing global exploration power of HS.

**Figure 6:**
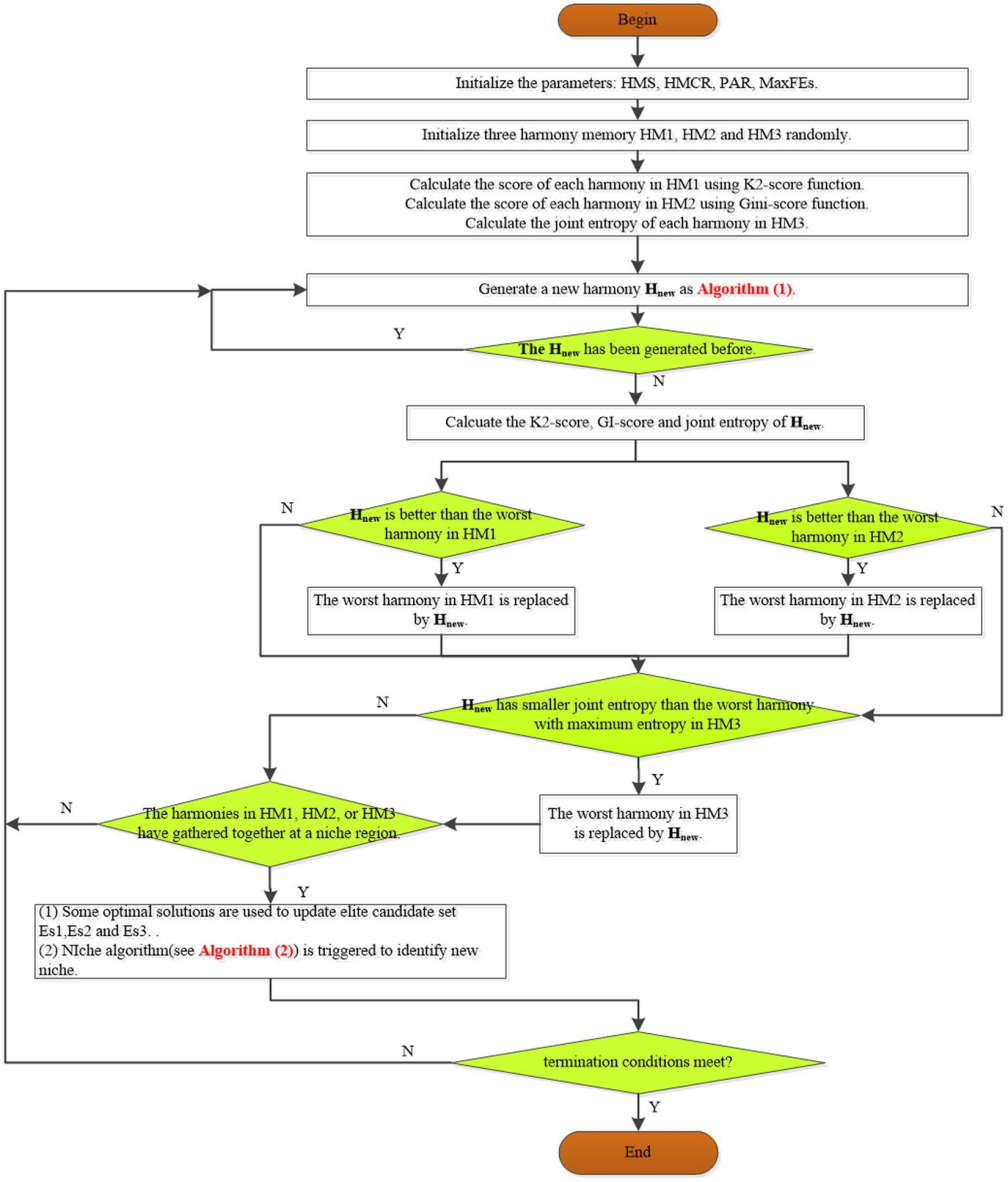
The flowchart of NHSA-DHSC for the first screening phase.

The flowchart of NHSA-DHSC for the first screening phase is shown in Fig. 6, where the algorithm (1) introduces the process for improvising a new harmony and algorithm (2) presents the method for identifying niche region.

Figure A–5 in Supplementary info file shows an example explaining the process of the NHSA-DHSC algorithm for detecting 3-way disease-causing models with a total SNP number of 10.

**Algorithm (1):**
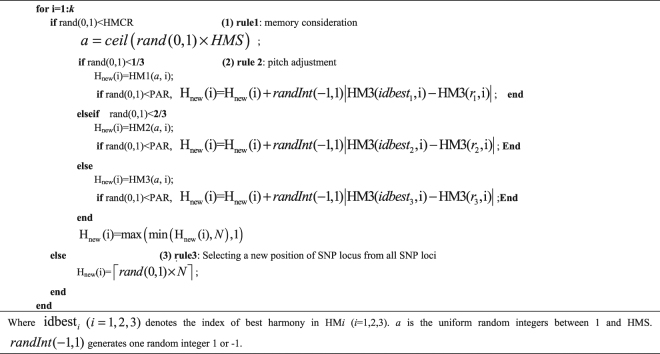
Generating a new harmony H_new_.

**Algorithm (2):**
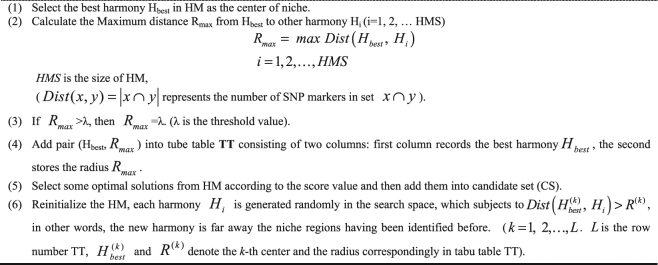
(Niche identification algorithm) During several iterations (T times), if there is no improvement for harmony memory (HM), we think all the solutions in HM might have been aggregated into a local region. And at this moment, niche identification algorithm is triggered to identify the niche, its work as follows:

In the search process of NHSA-DHSC, the niche identification algorithm is automatically triggered to identify a niche region when the harmony memories HM1, HM2 and HM3 cannot be updated during several iterations. Within a niche region, the radius of the niche is recorded for preventing generating new solution in the niche, which can effectively avoid the search algorithm trapping into a local region. In the supplementary info file, the niche technique is introduced in detail.

### Evaluation functions for calculating the association of SNP combinations with the phenotype

Three lightweight evaluation methods, Bayesian network based K2-score, Gini score and joint entropy, are adopted for improving detection power and speed of the HS algorithm, where the former two are for the adaptability of the diversity of disease models, and the latter is for the disease models with low marginal effect.

#### K2-score and GI-score

By for calculating the association of SNPs with the phenotype, Bayesian network based K2-score and Gini index (see supplementary info file) are first consideration. The two scoring methods are complementary for identifying diverse disease models^30^, some models that cannot be identified correctly by K2-score can be correctly identified by Gini-score and vice versa. Furthermore, as can be seen in equations (1–2), both K2-score and GI-score just only require calculating the number of genotype combinations once for each *k-way* SNP-combination, which are not repeatedly count the frequency of genotype combinations.

1 $$K2-Score=\prod _{i=1}^{I}(\frac{(J-1)!}{({n}_{i}+J-1)!}\prod _{j=1}^{J}{n}_{ij}!)$$

2 $$GI-score=\sum _{i=1}^{I}{P}_{i}\cdot (1-\sum _{j=1}^{J}{p}_{i,j}^{2})=\sum _{i=1}^{I}\frac{{n}_{i}}{n}\times (1-\sum _{j=1}^{J}{(\frac{{n}_{ij}}{{n}_{i}})}^{2})$$

Where ${p}_{i,j}$(*p*_*i*,*j*_ = *n*_*ij*_/*n*_*i*_) is the estimated probability that the *i* ^th^ genotype combination actually associated with phenotype ${y}_{j}$. $(1-\sum _{j=1}^{J}{p}_{i,j}^{2})$ is the estimated probability that genotype combination is misclassified as phenotype ${y}_{j}$. ${P}_{i}$(*P*_*i*_ = *n*_*i*_/*n*) is the percentage of *i* ^th^ genotype combination in sample set.

#### Joint entropy as a heuristic factor for guiding HS to explore the disease-causing SNP combinations with very low or even no marginal effect

In general, intelligent search algorithm (e.g. HS) can obtain the global optimization solution with a very small number of evaluating to the trail solutions. It is based on some heuristic factors available to speed up the search process. Similarly, for the detection of disease causes, the heuristic factors are also very important, such as marginal effects of individual SNP to phenotype, which can guide the HS to search the disease-causing loci quickly. Nevertheless, sometimes individual SNP site contained in a high-order disease-causing model has no marginal effect on phenotype, and available evaluation methods, such as Bayesian network, logistic regression, mutual information and many more, cannot distinguish whether a k-way SNP-combination contains portion of disease-causing SNP locus. In this scenario, a *k-way* disease-causing model is just as if an isolated point in a very large search space, none of heuristic factors (clues) can guide HS algorithm to find the disease-causing model quickly.

To address the problem, we did a great deal of experiments and found a *k-way* (k > 2) SNP-combination including part of disease-causing SNP markers often has smaller joint entropy than that including no SNPs in the causative combination model, which is because the genotype of disease-causing SNP-combinations often has smaller divergence than that of no disease-causing SNP-combinations. In other words, joint entropy has the capability of differentiating SNP combinations containing part of causative SNPs from the SNP combinations containing no causative SNPs. However, for the disease data with very low marginal effect, existing scoring methods usually have no ability to distinguish the SNP combination including some of risk factors from other SNP combination models, such as the Bayesian network based K2-score, logistic regression and Gini score.

In supplementary info file, we compare joint entropy with logistic regression based AIC-score and Bayesian network based K2-score^17^, the two compared scoring methods are very effective for detecting disease models with marginal effect, we can see from the results that the joint entropy is more effective for detecting high-order disease-causing models with very low marginal effect than Bayesian network based K2-score and logistic regression.

For a *k-way* SNP-combination $X=({X}_{1},{X}_{2},\cdots ,{X}_{k})$, its joint entropy can be calculated as follow

3 $$\begin{array}{rcl}H(X) & = & -\sum _{{x}_{1}\in {X}_{1}}\cdots \sum _{{x}_{n}\in {X}_{k}}p({x}_{1},{x}_{2},\cdots ,{x}_{k}){\mathrm{log}}_{2}p\,({x}_{1},{x}_{2},\cdots {x}_{k})\\  & = & -\sum _{i=1}^{I}{P}_{i}{\mathrm{log}}_{2}{P}_{i}=-\sum _{i=1}^{I}\frac{{n}_{i}}{n}{\mathrm{log}}_{2}\frac{{n}_{i}}{n}\end{array}$$

Where *n*_*i*_ is the number of samples in the dataset taking the *i* ^th^ genotype combination, *n* is the total number of samples in the dataset.

It can be seen obviously from equations (1), (2) and (3) that the k2-score, GI-score and joint entropy can be obtained simultaneously by calculating the genotype frequency on *k-way* SNP-combination, and the computation cost of the three scoring functions is also non-additivity, which means that we need not to repeatedly calculate the number of sample taking *i-*th genotype combination three times.

It’s important to note that the equation (3) considers only the genotype of SNP combination without regard to the phenotype. However, in equations (1) and (2), the genotype *X* and phenotype *Y* are considered simultaneously. In this work, the main role of joint entropy is rather than an identification method of disease-causing model, it is utilized as heuristic factor to guide the harmony search algorithm to rapidly locate the disease-causing SNP markers with no or very low marginal effect.

#### G-test

G-test is a maximum likelihood statistical significance test^31^. Compared to chi-squared test, the G-test will lead to the same test effect for samples of a rational size. However, for some cell case it is always better than the chi-squared test^56^. And for testing goodness-of-fit, G-test statistical method is more efficient than Pearson *χ* ^2^ test method^56–58^.

For *k-way* SNP combination model, the formula for calculating *G* value is as follow

4 $$G=2\sum _{i=1}^{I}\sum _{j=1}^{J}{Q}_{ij}\cdot {P}_{ij}$$

where, *O*_*ij*_ and *E*_*ij*_ are respectively the observed numbers and expected number of genotypes when phenotype takes the state *y*_*j*_ and genotypes take *i* ^th^ *k*-combination. The ln denotes natural logarithm function. We can get the observed number *O*_*ij*_ from dataset by using simple counting statistics method. The expected number *E*_*ij*_ of genotype frequency could be obtained according to Hardy-Weinberg principle^59^.

For high-order SNP combination, what often happens is that the number of some genotype combinations equals zero or very small, for example, a *2-way* SNP combination has nine 2-way genotypes as Table 5.

**Table 5:** Contingency table for 2-way SNP combination.

		2-way genotype								
		0–0	0–1	0–2	1–0	1–1	1–2	2–0	2–1	2–2
**Case**	**Observed number**	50	50	0	50	0	2	40	18	30
	**Expected number**	55	27.5	0.5	54	0	1	45	24.5	32.5
**Control**	**Observed number**	60	5	1	58	0	0	50	31	35
	**Expected number**	55	27.5	0.5	54	0	1	45	24.5	32.5

As shown in Table 5, there are very few samples on genotypes “0–2”, “1–1” and “1–2”. The conventional G-test method considers all the nine genotypes and the degree of freedom is equal to (2–1) × (9–1) = 8. In this work, we think the three columns (“0–2”, “1–1” and “1–2”) should not be considered, and the degree of freedom equals (2–1) × (6–1) = 5, which is more precise than the standard G-test method.

To enhance the statistical precision of G-test, we do a minor modification for calculating G-test value as follows,

$$G=2\sum _{i=1}^{I}\sum _{j=1}^{J}{Q}_{ij}\cdot {P}_{ij}$$

$${P}_{ij}=\{\begin{array}{c}\mathrm{ln}\,\frac{{Q}_{ij}}{{E}_{ij}},\sum _{j=1}^{J}Q{}_{ij}\, > \,\xi \\ 0,otherwise\end{array}$$

The degree of freedom *d* (*d* = (*I* − 1)(*J* − 1) is modified correspondingly, as follows:

$$\begin{array}{c}d=(I-1)(J-1)\\ for\,{\rm{i}}={\rm{1}}\to {\rm{I}}\\ \quad \quad {\rm{if}}\,\sum _{j=1}^{J}Q{}_{ij}\, < \,\xi \\ \quad \quad \quad d=d-1\\ \quad \quad {\rm{endif}}\\ {\rm{endfor}}\end{array}$$

### Simulation Datasets

#### Twelve disease models with marginal effects (DME)

The 12 DME models^17^ have both marginal effects and interaction effects, which contain four multiplicative models (DME-1~DME-4), four threshold models (DME 5- DME 6) and four concrete models (DME 7- DME 12).

DME-1~DME-4 (H^2^ = 0.005, MAF = 0.05, 0.1, 0.2 and 0.5) are multiplicative models with two disease locus, in which the disease prevalence given the frequency of genotype combination increases multiplicatively with the incremental presence of the disease. The genetic heritability (H^2^) of DME 1- DME 4 are all equal to 0.005, minor allele frequencies (MAF) of them equal 0.05, 0.1, 0.2 and 0.5, respectively. It is very difficult to identify the disease locus from the four DME models due to very low genetic heritability.

DME-5~DME-8 (H^2^ = 0.02, MAF = 0.05, 0.1, 0.2 and 0.5) are the threshold models in which the prevalence of genotype frequency does not increase until the number of disease alleles pass the threshold. The four DME models have strong marginal effect and interaction effect, in which a SNP marker with strong marginal effect would form many false disease models with other SNP markers that are not truly associated with the phenotype state.

DME-9~DME-12 (H^2^ = 0.02, MAF = 0.05, 0.1, 0.2 and 0.5) are the concrete model that has low marginal effect and strong interaction effect. Characteristics of these twelve DME models are compared in Figs. E-1~E-3 of supplementary info file, the parameters of 12 DME models are presented in Table E-1 (see supplementary info file).  For each DME model, there are 100 simulation datasets generated using GAMETES_2.0^60^ (https://sourceforge.net/projects/gametes/).

#### Eight high-order disease models with no marginal effects (DNME)

The DNME models are not constrained to specific predetermined models^61^. They are generated using multi-objective optimization algorithm that aims to maximize the joint effects of k-SNP, minimize the marginal effects of individual SNPs and limit to the Hardy-Weinberg equilibrium (HWE) constraints. The data sets of DNME models are downloaded from http://discovery.dartmouth.edu/model_free_data/, which contain 8 DNME data models (see Table E-2 in supplementary info file) with three to five functional SNPs. For each data model, there are 100 datasets each having 1500 controls and 1500 cases. The DNME-2, DNME-4 and DNME-6 are constrained by HWE; the other five are no HWE constraint.

#### Real AMD data

We use NHSA-DHSC algorithm to conduct high-order SNP association study on AMD data (Age-related macular degeneration)^33^. The AMD data contains 103611 SNPs genotyped for 50 controls and 96 cases. The experiment aims to find out all suspected high-order SNP combinations associated strongly with the phenotype.

### Evaluation metrics

In simulation experiments, we adopt seven indices (Runtime, Power, MEs, TPR, SPC, ACC, and FDR) to evaluate the performance of algorithms. The seven indices are defined as follows,

1. Runtime: The time it takes for finding a disease-causing model from beginning search to the end.
2. Power = #S/#T. Power is a measure of the capability for detecting the disease-causing models from all dataset, where the **#S** is the number of having found the disease-causing model from all **#T** dataset (in the experiment, there are 100 data matrix for each disease model).
3. MEs denotes the mean number of SNP-combinations that need be calculated the association with phenotype using scoring methods before the disease-causing model is found. In the experiment, if the known disease-causing models have been found, the searching algorithm would be terminated automatically ahead of meeting termination condition, the number that *k-way* SNP combination models have been evaluated currently is defined as mean evaluation times (MEs) and the elapsed time from start to end is denoted as computation time (Runtime). The search algorithm would be terminated if the number of SNP combinations that are evaluated using evaluation functions is larger than maximum allowable number of times.
4. True positive rate: $\mathrm{TPR}=\mathrm{TP}/(\mathrm{TP}+\mathrm{FN})$
5. Specificity: $\mathrm{SPC}=\mathrm{TN}/(\mathrm{FP}+\mathrm{TN})$ (if FP+TN = 0, then SPC = 0)
6. Accuracy: $$\mathrm{ACC}=(\mathrm{TP}+\mathrm{TN})/(\mathrm{TP}+\mathrm{TN}+\mathrm{FN}+\mathrm{FP})$$
7. False discovery rate: $\mathrm{FDR}=\mathrm{FP}/(\mathrm{TP}+\mathrm{FP})$ (if TP + FP = 0, then FDR = 0)

The TPR, SPC and ACC in this study are employed to measure the statistical precision of the hypothesis testing method for having found disease-models in the screening stage. The TP is equal to the number of disease-models that have passed the threshold (Bonferroni correction, p-value = 0.05/N, N is the number of combinations) of the testing method, FN is the number of disease-models failed to pass the threshold of the testing method. FP is the number of non-disease-models passed the threshold, TN equals the number of non-disease-models failed to pass the threshold.

### Parameters setting of NHSA-DHSC

#### Experiments for simulation datasets

The parameters of NHSA-DHSC are set as follows:

The sizes of HM1, HM2, HM3, Es1, Es2 and Es3 are all equal to 50 for dataset with 100SNPs and 100 for dataset with more than 100SNP sites, the maximal size of candidate set (CS) is 10. HMCR = 0.9 and PAR = 0.35. In the second stage, the threshold of p-value equals 0.05/N (Bonferroni correction, N is the number of combinations). In order to prevent from the preference of location, we randomly embed the locations of disease-causing SNPs into the simulation data.

For CSE, the fraction of eggs discarded each generation is set to 0.25, maximum number of steps to take in a levy flight is set to 1, the number of groups is 10, and the number of nests is set to100.

The parameters of MACOED are set as: the number of ants is 500 for dataset with 1000SNPs, and 50 for dataset with 100SNPs.

For SNPHarvester, the maximal and minimal order of interactions is equal to *k* (for *k-way* models).

The parameters setting for Boost and Beam are set to the default value of original papers.

To make a fair comparison, for three intelligent search algorithms NHSA-DHSC, CSE and MACOED, we set the same terminal condition (Maximum number of evaluating the SNP-combinations: T_max_) T_max_ = 4500 for dataset with 100 SNP sites, T_max_ =60000 for 1000 SNPs.

We set the same computation environment for six compared algorithms: all experiments were performed on Windows 7 operation system with Intel(R) Core(TM) i3–3470 CPU@3.2 GHz, 8 GB memory, and all the program codes were written in MATLAB R2014b.

#### Experiments for AMD data

The sizes of HM1, HM2, and HM3 are set to 500.

The sizes of Es1, Es2, and Es3 are all equal to 2000.

HMCR = 0.9 and PAR = 0.35.

T_max_ = *k*×3E+6. (*k* is the number of SNP sites of high-order SNP combinations)

The experimental environment is the same as that of simulation dataset.

### Future work

It has been widely acknowledged that multiple SNP loci may be an important contributor to pathogenic factors of complex disease, however, at present there is still no an effective approach in detecting multi-loci disease-causing models at GWAS due to enormous computation burden. Therefore, detecting high-order disease models has many rooms to explore using high-performance and cloud computing. In addition, with the rapid development of new gene sequencing technique, detecting the epistatic interactions in non-coding genomic regions^62, 63^ and making sense of the rare variants at GWAS are worth to study in the future.

## Electronic supplementary material

Supplementary info

Dataset 1

Dataset 2

source codes of NSHA-DHSC
