# Author Correction: Niche harmony search algorithm for detecting complex disease associated high-order SNP combinations

**DOI:** 10.1038/s41598-018-24597-4

**Published:** 2018-04-17

**Authors:** Shouheng Tuo, Junying Zhang, Xiguo Yuan, Zongzhen He, Yajun Liu, Zhaowen Liu

**Affiliations:** 1 School of Computer Science and Technology, Xidian University, Xi’an, 710071 P.R. China; 2 School of Mathematics and Computer Science, Shaanxi University of Technology, Hanzhong, 723000 P.R. China

Correction to: *Scientific Reports* 10.1038/s41598-017-11064-9, published online 14 September 2017

The Acknowledgements section in this Article is incomplete.

“This work was supported by the Natural Science Foundation of China under Grants 61571341, 61201312, 91530113 and 11401357, Research Fund for the Doctoral Program of Higher Education of China (No. 2013 0203110017), the Fundamental Research Funds for the Central Universities of China (Nos BDY171416 and JB140306), the Natural Science Foundation of Shaanxi Province in China (2015JM6275), Free exploration projects for 2017 basic research-related expenses.”

should read:

“This work was supported by the Natural Science Foundation of China under Grants 61571341 and 11401357, the Fundamental Research Funds of China for the Central Universities (Nos JBZ170301 and 20101164977).”

