# Supplementary material for: Niche harmony search algorithm for detecting complex disease associated high-order SNP combinations
Source: Sci Rep. 2017 Sep 14;7:11529. doi: 10.1038/s41598-017-11064-9 (PMC5599559; doi:10.1038/s41598-017-11064-9)
Supplement: Supplementary file 1 — Supplementary info [file 41598_2017_11064_MOESM1_ESM.pdf]

Niche harmony search algorithm for detecting complex disease associated high-order SNP combinations

Shouheng Tuo[1, 2], Junying Zhang[1*], Xiguo Yuan[1], Zongzhen He [1], Yajun Liu[1], Zhaowen Liu[1]
1. School of Computer Science and Technology, Xidian University, Xi'an, 710071, P.R.China
2. School of Mathematics and Computer Science, Shaanxi University of Technology, Hanzhong 723000, P.R.China
* Corresponding Author: Junying Zhang (Email: jyzhang@mail.xidian.edu.cn, tuo_sh@126.com)

1 Niche harmony search algorithm

1.1 Standard Harmony Search Algorithm SNP

Harmony search (HS) algorithm is a swarm intelligent optimization algorithm (Z.W. Geem, J. Kim, J. Kim, G. Loganathan, 2001; Z.W. Geem, 2008)[1][2]. It mimics the process of improvising a musical harmony when a music orchestra is aiming at composing the most harmonious melody. For an optimization task, each harmony corresponds to a vector which consists of *k* decision variables. The harmony memory (HM) which consists of HMS harmonies is similar to the population in genetic algorithm (GA), where HMS is called the size of HM. In genome-wide association study (GWAS), we can treat a *k-way* interaction model as a harmony and our goal is to find a best harmony which has strongest association with phenotype. The optimization problem for *k-way* interaction model can be expressed as

$$\max_{X} f(X), X = (x_1, x_2, \cdots x_k) \tag{1}$$

where, $x_i \in \{1, 2, \cdots, N\}$, N is the number of SNPs. if $i \neq j$, then $x_i \neq x_j$.

The steps of harmony search algorithm are as follows:

Step 1. Parameter initialization.

The control parameters of harmony search algorithm are specified, which include HMS, harmony memory considering rate (HMCR), pitch-adjusting rate (PAR), fret width (*fw*) (fret width is called formerly bandwidth: *bw*) and the termination criterion (i.e., the maximum function evaluation times(MaxFEs)).

Step 2. Initializing the harmony memory (HM) and calculating the fitness value of each harmony.

```
For i=1: HMS
    X^i = ∅ ;
    For j=1: k
        Do
            a = ⌈rand(0,1) × N⌉ ;
        While ( a ∈ X^i )
        X^i ← a ;
    End
    // calculating the fitness value
    Score(i) = f(X^i) ;
End
```

where, rand(0,1) represents a uniformly distributed random number between 0 and 1.

The harmony memory (HM) consists of HMS harmonies, as follow,

$$\mathbf{HM} = \begin{bmatrix} \boldsymbol{X}^1 \\ \boldsymbol{X}^2 \\ \vdots \\ \boldsymbol{X}^{\mathrm{HMS}} \end{bmatrix} = \left[\begin{array}{cccc|c} x_1^1 & x_2^1 & \cdots & x_k^1 & Score(1) \\ x_1^2 & x_2^2 & \cdots & x_k^2 & Score(2) \\ \vdots & \vdots & \vdots & \vdots & \vdots \\ x_1^{\mathrm{HMS}} & x_2^{\mathrm{HMS}} & \cdots & x_k^{\mathrm{HMS}} & Score(\mathrm{HMS}) \end{array}\right]$$

Step 3. Improvising a new harmony $\boldsymbol{X}^{new}$.

$X^{new} = \varnothing$
For j=1:***k***
If *rand (0,1)*<HMCR
$$X^{new}(j) = \mathrm{HM}\left(\lceil rand(0,1) \times \mathrm{HMS} \rceil, j\right)$$
If *rand(0,1)*<PAR
$$X^{new}(j) = \lceil X^{new}(j) \pm fw \rceil$$
End
Else
$$X^{new}(j) = \lceil rand(0,1) \times N \rceil \;;$$
End
End

Step 4. Update operation: updating the worst harmony in HM.

If $Score(\boldsymbol{X}^{new}) > Score(\boldsymbol{X}^{idworst})$
$X^{idworst} = X^{new}$;
$Score(idworst) = f(X^{new})$;
End

where $idworst$ is the index of the worst harmony in HM.

Step 5. Checking the stopping criterion. If stopping criterion (MaxFEs) is meet, computation is terminated. Otherwise, Step 3 and Step 4 are repeated.

1.2 Niche technique

Niche technique, inspired by the way organisms evolve in nature [3], can be employed to enhance the population diversity and maintain multiple different optima in the search landscape [4]. For example, continual function schwefel 2.26 has many local optimal solutions, whose unique globally optimal solution (420.9867, 420.9867,…, 420.9867) is near the boundary (see **Fig.A-1**); the swarm intelligent search algorithm is very easy to trap into one of local optimal regions, which might cause the global optimal solution lost. In niche technique, when many candidate solutions are gathered together in a small region of search space, the niche algorithm is triggered to recognize the niche region, and then the recognized niche region is set as forbidden zone for forcing search algorithm to explore new solutions in unexplored regions. In **Fig.A-1**, red ellipse denotes a niche region, where c represents the center of this niche, two dimensional vector (r_1, r_2) is the radius of the niche region. Subsequently, the search algorithm explores new solutions only on the outside of the identified niche regions.

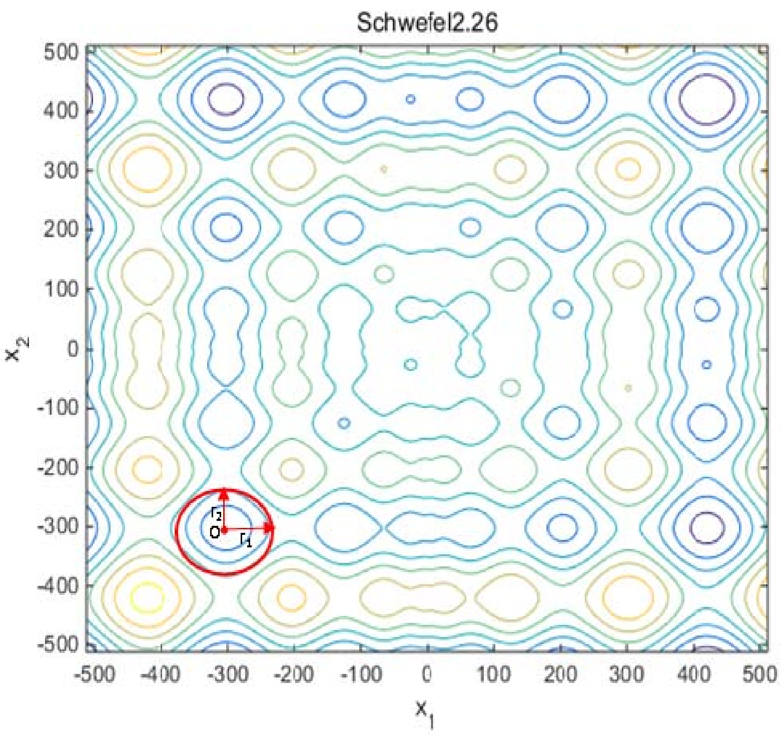

Fig.A-1 contour graph of schwefel 2.26. The schwefel2.26 is a complex multimodal function, which has many local minimum. In the fig, the region of red ellipse is a local region called niche. The center of the niche is in the position O, and its radius is (r1, r2).

1.3 Niche technique for discovering more disease factors

For a new complex disease, we previously have not known biological information, whose risk factors may be some single SNP locus, combinations of multi-SNP loci, or hybrid models composed of the additive models, epistasis models and so on.

To be able to detect various risk factors, we need consider as many kinds of SNP combination models as possible. For the disease models with strong marginal effects, it can be found easily and quickly using swarm intelligent algorithm, however, for datasets including multiple disease models with marginal effect and disease models without marginal effect, it is extremely difficult to detect all disease-causing models due to the interference with one another seriously.

To tackle this problem, in this study, a new niche technique is merged into harmony search algorithm for exploring all disease-causing SNP combinations efficiently. The goal of the niche technique is mainly to discover some SNP-combinations with strong marginal effect, where the marginal effects do not only come from single SNP markers, but also may be synergistic effects of multi-SNP makers. In the search process of HS, the position and size of each niche region are recorded into a tabu table for forcing the HS algorithm to search new solutions in unexplored regions. In this way, all possible *k-way* SNP combination models having strong association with phenotype can be extracted one by one.

For example, a ***k**-way* disease-causing model $(x_1, x_2, \cdots, x_k)$(x_i denotes the position index of ith SNP marker in dataset) contains a *2-way* sub-model (x_1, x_2) that has strong combination marginal effect to phenotype. After a period of HS search, many *k-way* models containing the sub-model (x_1, x_2), such as $(x_1, x_2, x_{s3}, x_{s4}, \cdots, x_{sk})$ $(x_i \neq x_j, i \neq j)$, might have been attracted into harmony memory (HM1, HM2, or HM3), which usually have higher score (K2-score, Gini-score or joint entropy)

than the models without containing the sub-model (x_1, x_2). In this case, the HS is very easily trapped into local search and hard to jump out of the local region for exploring new disease-causing models due to the strong attraction of the marginal effect. At this point, the niche algorithm is triggered automatically to identify niche region whose center c has the highest score (e.g. $(x_1, x_2, \cdots, x_k)$) and radius r is defined as the number of common SNP loci of all *k-way* models in HM, (in this example, there are two the common SNP loci x_1 and x_2, thus $r = k-2$).

When niche region (c, r) have been identified using the niche algorithm (see the identification algorithm(1)), it will be stored into tabu table (TT) that is used for avoiding evaluating some models repeatedly and forces the HS algorithm to search other regions for finding new possible risk factors. Then some best solutions (having strongest causal association with phenotype status) which are chosen from HM1, HM2 and HM3 are put into elite solution set Es1, Es2 and Es3, respectively. After the process HS search is end, the Es1, Es2 and Es3 are combined into a candidate set (CS).

Fig.A-2 (a) and **Fig.A-2** (b) show the landscape of Bayesian score of all 2-*way* SNP combinations to phenotype. As can be seen from **Fig.A-2** (a) that there are two 2-*way* disease-causing models (30, 70) and (10, 50), where the model (30, 70) has stronger marginal effect than (10, 50). In the searching process of HS algorithm, the populations are easily gathered around (30, 70) owing to the strong marginal effect and result in a failed search for the model (10, 50). For this case, we use niche technique to overcome the local search problem. If the populations (HM1, HM2 and HM3) of HS cannot be updated during a period of searching time, the niche technique will be triggered to identify the niche region $(c, r), (c = (30, 70), r = 1)$. Then the two SNP loci 30 and 70 are culled from data. **Fig.A-2** (b) is the surface of the remaining data, from this it indicates that the 2-*way* model (10, 50) has more obvious attraction than other loci. Subsequently, the model (10,50) is easily found by the HS algorithm.

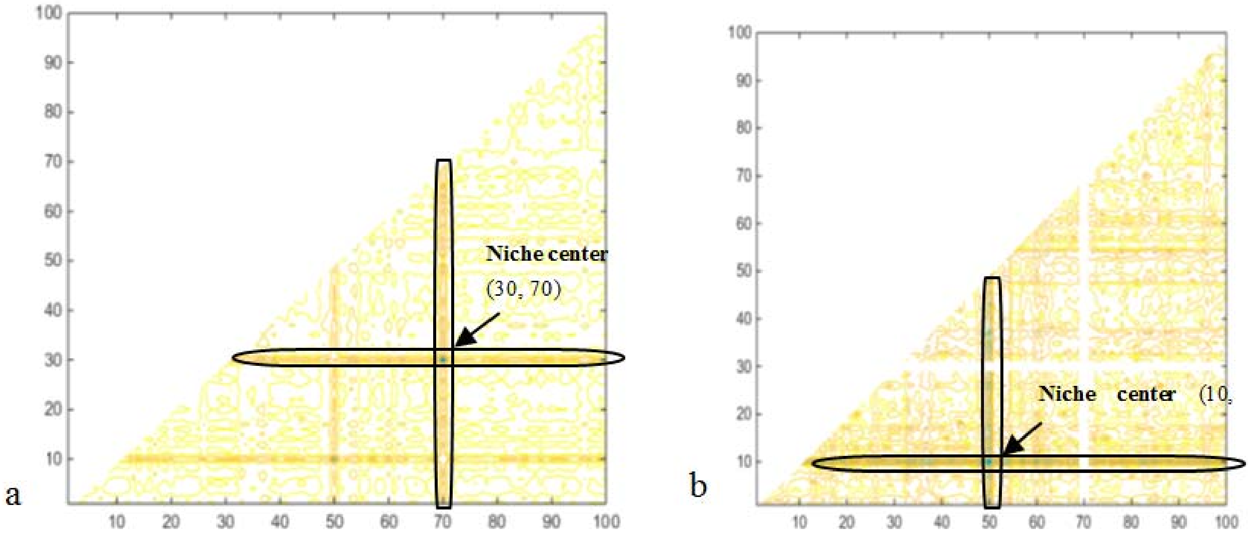

Fig.A-2 contour graph of scoring function for 2-*way* SNP combination models. In this figure, there are two 2-*way* disease-causing models (30, 70) and (10, 50) which both have marginal effect to phenotype. The marginal effect of disease model (30, 70) forms a niche region {(30, *), (70,*)} (* represents any SNP locus). (10, 50) also form a niche region {(10, *), (50,*)}

In the search process of NHSA, the niche identification algorithm is automatically triggered to identify a niche region when the harmony memories HM1, HM2 and HM3 cannot be updated during several iterations. Within a niche region, the radius of the niche is recorded for preventing generating new solution in the niche, which can effectively avoid the search algorithm trapping into a local region.

Algorithm (1): Niche identification algorithm:

During several iterations (T times), if there is no improvement for harmony memory (HM), we think all the solutions in HM might have been aggregated into a local region. And at this moment, **niche identification algorithm** is triggered to identify the niche, its work as follows:

(1) Select the best harmony H_{best} in HM as the center of niche.

(2) Calculate the Maximum distance R_{max} from H_{best} to other harmony H_i (i=1, 2, … HMS)

$$R_{max} = max\ Dist\left(H_{best},\ H_i\right)$$
$$i = 1, 2, \ldots, HMS$$

HMS is the size of HM,

($Dist(x, y) = |x \cap y|$ represents the SNP number of set $x \cap y$).

(3) If R_{max} >λ, then R_{max} =λ. (λ is the threshold value).

(4) Add pair (H_{best}, R_{max}) into tube table **TT** that consists of two columns: first column records the best harmony H_{best}, the second stores the radius R_{max} .

(5) Select some optimal solutions (according to the score value) from HM and add them into elite candidate set (ECS).

(6) Reinitialize the HM, each harmony H_i is generated randomly in the search space, which subjects to $Dist\left(H_{best}^{(k)},\ H_i\right) > R^{(k)}$, in other words, the new harmony is far away the niche regions having been identified before. ($k = 1,\ 2, \ldots, L$. L is the row number TT, $H_{best}^{(k)}$ and $R^{(k)}$ denote the *k*-th center and the radius correspondingly in tabu table TT).

2 Bayesian network scoring criteria and Gini scoring criteria

2.1 K2-Score

(1) Bayesian model

A Bayesian network (BN) is a type of statistical model (probabilistic directed acyclic graphical model) which represents a set of random variables and their conditional dependencies by using a directed acyclic graph (DAG) (http://en.wikipedia.org/wiki/Bayesian_network). In the DAG, Nodes denote random variables, and the edges represent conditional dependences between two linked nodes.

There are more than ten kinds of BN models [**5-7**] that have been developed to find causal relationships, perform explanatory analysis , describe the causal influence and make predictions. In GWAS studies, BN model is also used to detect the interaction effect among SNP markers, which can represent the relationship between genetic variants and disease status.

Let a set of SNP variables $X = \{x_1, x_2, \cdots, x_N\}$ indicate *N* SNP markers for *S* individuals, phenotype variable be Y with values $(y_1, y_2, \cdots, y_J)$; we represent the homozygous major allele, heterozygous allele and homozygous minor allele as 0, 1 and 2. In a DAG of Bayesian network for representing the relationships of SNP markers and disease states, there are only directed edges linking from the SNP markers to diseases status, and there is no any edge linked from disease state to SNP markers and no edge connected among SNP markers. In the DAG, if and only if SNP x_i is a direct cause of phenotype y_j, there is a directed edge linking from node x_i to node y_j. **Figure A-3** shows an example of *k*-SNPs epistasis Bayesian network model associated with phenotype y_j, where $c_i = (x_{i_1}, x_{i_2}, \cdots, x_{i_k})$ is a *k*-combination of genotypes (*k* SNPs markers), $y_j (0 \le j \le J)$ denotes the phenotype state.

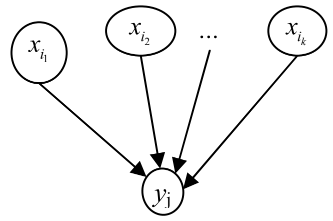

Fig.A-3 *k*-SNPs epistasis Bayesian network model

A *k*-combinatorial model associated with phenotype can be expressed by the probability distribution [7] as follow:

$$P(Y \mid c_i) = P(Y \mid x_{i_1}, x_{i_2}, \cdots, x_{i_k}) \quad (2)$$

where, *Y* is the state variable of phenotype and ($x_{i1}, x_{i_2}, \ldots, x_{i_k}$) a *k*-combination of genotype variables. For a *k*-SNPs combinational model associated with phenotype, there are k^3 possible genotypic combinations for diploid genomes (each SNP markers has three values: 0, 1 and 2) and each genotypic combination is associated with a probability distribution over the phenotype variable *Y*.

There have been several Bayesian network structure learning models successfully used to identify the SNP interaction, such as score-based methods used to evaluate the association between genotypes and phenotype by scoring the model's fitness to the data.

(2) Bayesian Scoring (K2-Score)

The Bayesian theorem can be used to evaluate the fitness of any given combinatorial model. Let a sample dataset *D* that contains values for SNPs and phenotype states, the BN model *M* can be expressed as the following equation:

$$P(M \mid D) = \frac{P(D \mid M)P(M)}{P(D)} \quad (3)$$

where, $P(M \mid D)$ is the posterior probability given sample dataset *D*, $P(M)$ is the prior probability of *M*, $P(D \mid M)$ denotes the marginal likelihood. *P(D)* represents the probability of the data *D* , which is a constant for all Bayesian network models based on data set *D* [8]. Thus, the equation (3) can be written as

$$P(M \mid D) \propto P(D \mid M)P(M) \quad (4)$$

where, the symbol " $\propto$ " denotes that the $P(M \mid D)$ is proportional to $P(D \mid M)P(M)$.

$P(D \mid M)$ can be written as follow,

$$P(D \mid M) = \int_{\theta_M} P(D \mid M, \theta_M)P(\theta_M \mid M)d\theta_M \quad (5)$$

where, θ_M denotes the parameters of the probability distributions of phenotype variable *Y* given the SNP variables $\{x_1, x_2, \cdots, x_N\}$.

According to the theorem 1 given in [8] , under the following assumptions:

(1) The values of the phenotype variable are generated via independent identically distributed (i.i.d) sampling from P(*Y* |*X*).

(2) Prior belief about the distribution *P(Y|X=x_i)* is independent of prior belief about *P(Y|X=x_j)* for all values x_i and x_j of *X* $(x_i \neq x_j)$.

(3) For all values x_j, prior belief about the distribution *P(Y|X=x_j)* is modeled using Dirichlet dirtribution with hyperparameters α_i and α_{ij} .

The equation (5) has a closed-form solution which is as following expression:

$$P(D \mid M) = \prod_{i=1}^{I} \left(\frac{\Gamma(\alpha_i)}{\Gamma(n_i + \alpha_i)} \prod_{j=1}^{J} \frac{\Gamma(n_{ij} + \alpha_{ij})}{\Gamma(\alpha_{ij})} \right) \quad (6)$$

where, *I* is the number of genotype combinations (there are 3^k SNP markers' combinations if *k*-SNP nodes are connected to the

phenotype y_j) , J is the number of phenotype states Y (which is equal to two for case-control dataset). Γ is the gamma function, n_i is the number of cases in the dataset with SNP nodes taking the i-th genotype combination, n_{ij} represents the number of cases that belong to the phenotype state j where the k-SNPs variables have i-th genotype combination. α_{ij} are the hyperparameters in a Dirichlet distribution [7,9] which define the prior probability over the the parameters θ_M , and $\alpha_i = \sum_{j=1}^{J} \alpha_{ij}$. We can view the hyperparameters as prior belief numbers of cases from a previous hypothetical study which belongs to the disease state j where the genotypes is given by *i-th* combination. When parameters $\alpha_{ij} > 0$ *and* $n_{ij} > 0$, *the* Equation (6) can be expressed as follow:

$$P(D \mid M) = \prod_{i=1}^{I} \left(\frac{(\alpha_i - 1)!}{(n_i + \alpha_i - 1)!} \prod_{j=1}^{J} \frac{(n_{ij} + \alpha_{ij} - 1)!}{(\alpha_{ij} - 1)!} \right) \tag{7}$$

According to the analysis in [7], for the BN model M, it is unknown for us to prior belief of the model M. Thus, we can consider all BN models to be equally plausible and think the prior probability $P(M)$ is a constant for all BN models. Thereby, the Equation (4) can be expressed as :

$$P(M \mid D) \propto P(D \mid M) \tag{8}$$

According to Equation (7).

$$P(M \mid D) \propto \prod_{i=1}^{I} \left(\frac{(\alpha_i - 1)!}{(n_i + \alpha_i - 1)!} \prod_{j=1}^{J} \frac{(n_{ij} + \alpha_{ij} - 1)!}{(\alpha_{ij} - 1)!} \right) \tag{9}$$

Set all $\alpha_{ij} = 1$ ($\alpha_i = \sum_{j=1}^{J} \alpha_{ij} = J$), which means that all possible distributions of Y given $X = x_i$ is equally likely. Thus the Equation (9) can be written as

$$P(M \mid D) \propto \prod_{i=1}^{I} \left(\frac{(J-1)!}{(n_i + J - 1)!} \prod_{j=1}^{J} n_{ij}! \right) \tag{10}$$

The evaluation criterion of BN model turns to the K2-Score as follow formula

$$K2 - Score = \prod_{i=1}^{I} \left(\frac{(J-1)!}{(n_i + J - 1)!} \prod_{j=1}^{J} n_{ij}! \right) \tag{11}$$

2.2 Gini Index

For a binary classification case-control problem, the Gini index is a diversity index [11] which is defined as Equation (12)

$$Gini - score = \sum_{i=1}^{I} P_i \cdot \left(1 - \sum_{j=1}^{J} p_{i,j}^2 \right) \tag{12}$$

where, $p_{i,j}$ is the estimated probability that the i-th genotype combination actually associated with phenotype y_j . $\left(1 - \sum_{j=1}^{J} p_{i,j}^2 \right)$ means the estimated probability that genotype combination is misclassified as phenotype y_j . P_i is the percentage of i-th genotype combination in sample set.

For example, in Table A-1, there are two SNP combination: (x_1 x_2 x_3) and (x_1 x_4 x_7). The computational method of impurity of Gini index is as follow.

Table A-1. Data set of two SNP combinations and disease state

Sample id	*c*	$x_1\,x_2\,x_3$	y	*Sample id*	*c*	$x_1\,x_4\,x_7$	y
1	c_1	*001*	1	1	c_1	011	0
2	c_2	101	0	2	c_3	100	1
3	c_3	110	1	3	c_2	102	1
4	c_3	110	1	4	c_3	100	0
5	c_2	101	1	5	c_2	102	0
6	c_3	110	1	6	c_2	102	1
7	c_4	201	0	7	c_3	100	0
8	c_4	201	0	8	c_3	100	1

$$P_1 = \frac{1}{8} = 0.125, P_2 = \frac{2}{8} = 0.25, P_3 = \frac{3}{8} = 0.375, P_4 = \frac{2}{8} = 0.25$$

$$p_{1,0} = \frac{0}{1} = 0, \quad p_{1,1} = \frac{1}{1} = 1$$

$$p_{2,0} = \frac{1}{2} = 0.5, \ p_{2,1} = \frac{1}{2} = 0.5$$

$$p_{3,0} = \frac{0}{3} = 0, \quad p_{3,1} = \frac{3}{3} = 1$$

$$p_{4,0} = \frac{2}{2} = 1, \quad p_{1,1} = \frac{0}{2} = 0$$

$$\begin{aligned}\Phi\left(L(x_1x_2x_3)\right) &= 0.125\times(1-1) \\ &\quad +0.25\times\left(1-(0.25+0.25)\right) \\ &\quad +0.375\times(1-1) \\ &\quad +0.25\times(1-1) \\ &= 0.125\end{aligned}$$

$$\begin{aligned}\Phi\left(L(x_1x_4x_7)\right) &= 0.125\times(1-1) \\ &\quad +0.375\times\left(1-\left(\frac{1}{9}+\frac{4}{9}\right)\right) \\ &\quad +0.5\times\left(1-\left(\frac{9}{16}+\frac{1}{16}\right)\right) \\ &= 0.354167\end{aligned}$$

2.3 Comparison joint entropy with other scoring functions

In a ***k-way*** SNP causative genotype combination $(x_1, x_2, \cdots, x_k)$, even though single SNP locus $x_i (i = 1,2,\cdots,k)$ has no marginal effect to phenotype status, its an ***s-way*** sub-genotype combination $(x_{m_1}, x_{m_2}, \cdots, x_{m_s})$ $(m_j = 1,2,\cdots,k, s < k)$ may be likely to have marginal effect to phenotype, and the ***s-way*** sub-genotype combination has smaller dispersity than an ***s-way*** random genotype combination.

For example, a SNP dataset with 3000 rows (sample number) and 100 columns (number of SNPs) is composed of 1500 case sample and 1500 control sample, where 100 SNP loci are denoted as $(x_1, x_2, \cdots, x_{96}, x_{97}, \cdots, x_{100})$. In the dataset, the 5-*way* SNP combination $(x_{96}, x_{97}, \cdots, x_{100})$ is the disease-causing interaction model and each SNP loci has not marginal effects to phenotype. The corresponding dataset of this disease mode comes from http://discovery.dartmouth.edu/model_free_data/ (fivewayNoLowBests) [10], which disables the discovery of disease-causing models for some existing heuristic methods owing to lacking the clues of causative SNP markers. For each proper subset of $\{x_{96}, x_{97}, \cdots, x_{100}\}$, its *k-way* (*k*<5) SNP combinations has not significant association with phenotype if we use the traditional evaluation methods (K2-score, Logistic regression [9], mutual information and so on), which cannot differentiate from other *k-way* combinations on all SNP loci. However, the joint entropy of

genotype combination, without considering phenotype, can differentiate the proper subset of $\{x_{96}, x_{97}, \cdots, x_{100}\}$ from other identical size of genotypes that are the combinations of other SNP loci.

We compared joint entropy with logistic regression based AIC-score [9] and Bayesian network based K2-score by calculating the association between each ***2-way*** SNP genotype combinations and phenotype outcome. The evaluation scores of three methods for all ***2-way*** genotype combinations are displayed respectively with surface graph in **Fig.A-4**(a-c), and the corresponding contour plots are shown in **Fig.A-4**(d-f).

As shown in **Fig.A-4** (a-b) and **Fig.A-4** (d-e), the logistic-regression-based AIC-score criterion and Bayesian-network-based K2-score criterion have hardly any discriminability for different ***2-way*** genotype combinations. Yet, in **Fig.A-4** (c) and **Fig.A-4** (f), the joint entropies of sub-genotype combination that is combined with part of SNP markers $\{x_{96}, x_{97}, \cdots, x_{100}\}$ are obvious smaller than that of other sub-genotype combinations which contain none of SNP markers $(x_{96}, x_{97}, \cdots, x_{100})$. We also investigate the ***3-way*** and ***4-way*** genotype that contain several disease-causing SNP markers $(x_{96}, x_{97}, \cdots, x_{100})$ on eight datasets. The investigate results indicate that the genotype combinations including several disease-causing SNP markers have smaller value of joint entropy than that of random genotype combinations.

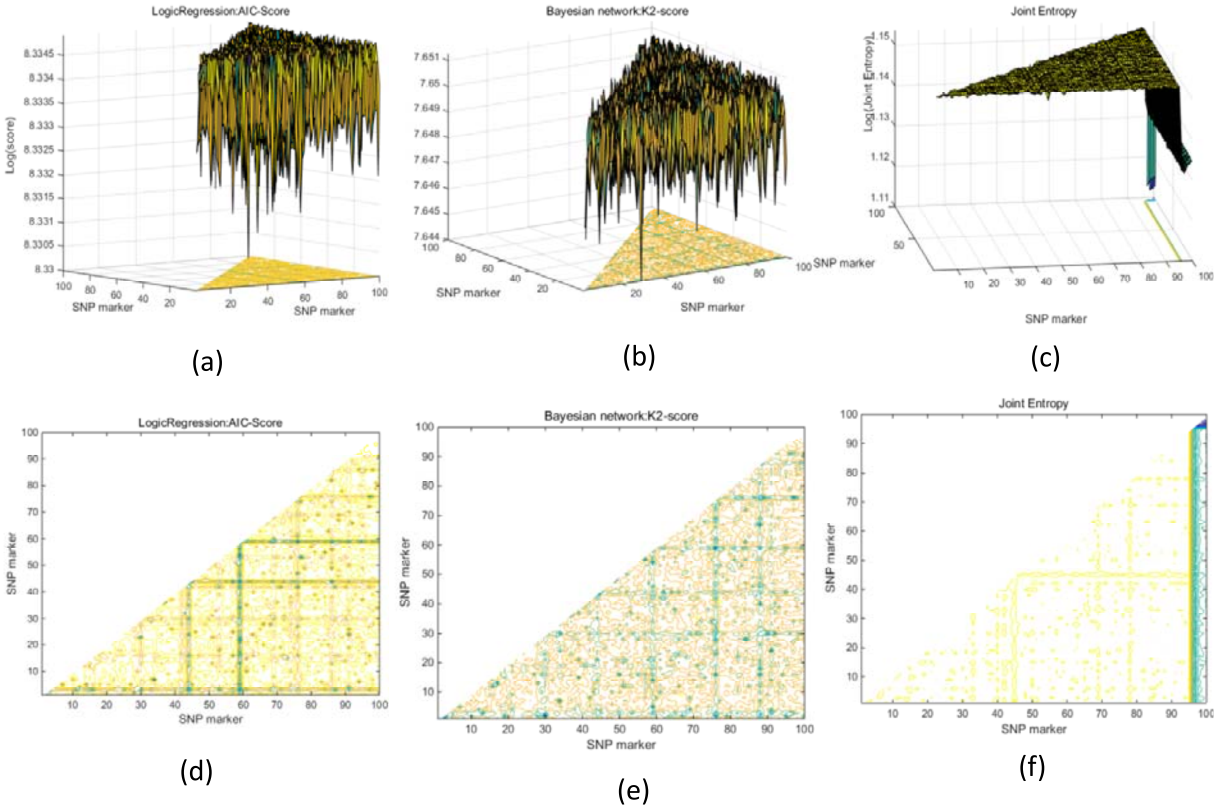

Fig.A-4 Comparison of three Evaluation Indexes. Fig (a), (b) and (c) are the scoring surface graphs for all *2-way* SNP combination models evaluated using logistic regression based AIC criterion, Bayesian network based K2-score and joint entropy, respectively. The smaller the score is, the higher the association with phenotype. In **Fig (a)** and **(b)**, the distribution of scores is **disorderly and unsystematic**; it is very difficult to discover heuristic information from them to explore the disease-causing SNP combination models. Yet, the joint entropy, in **Fig. c**, that only considers the genotype combinations without consideration of phenotype, display **obvious discriminatio**n between disease-causing SNP markers

$(x_{96}, x_{97}, \cdots, x_{100})$ and other markers. **Fig (d), (e) and (f)** are the contour graphs for all 2-*way* SNP combination models evaluated using logistic regression based AIC criterion, Bayesian network based K2-score and joint entropy, respectively.

3 The process of the NHSA-DHSC algorithm

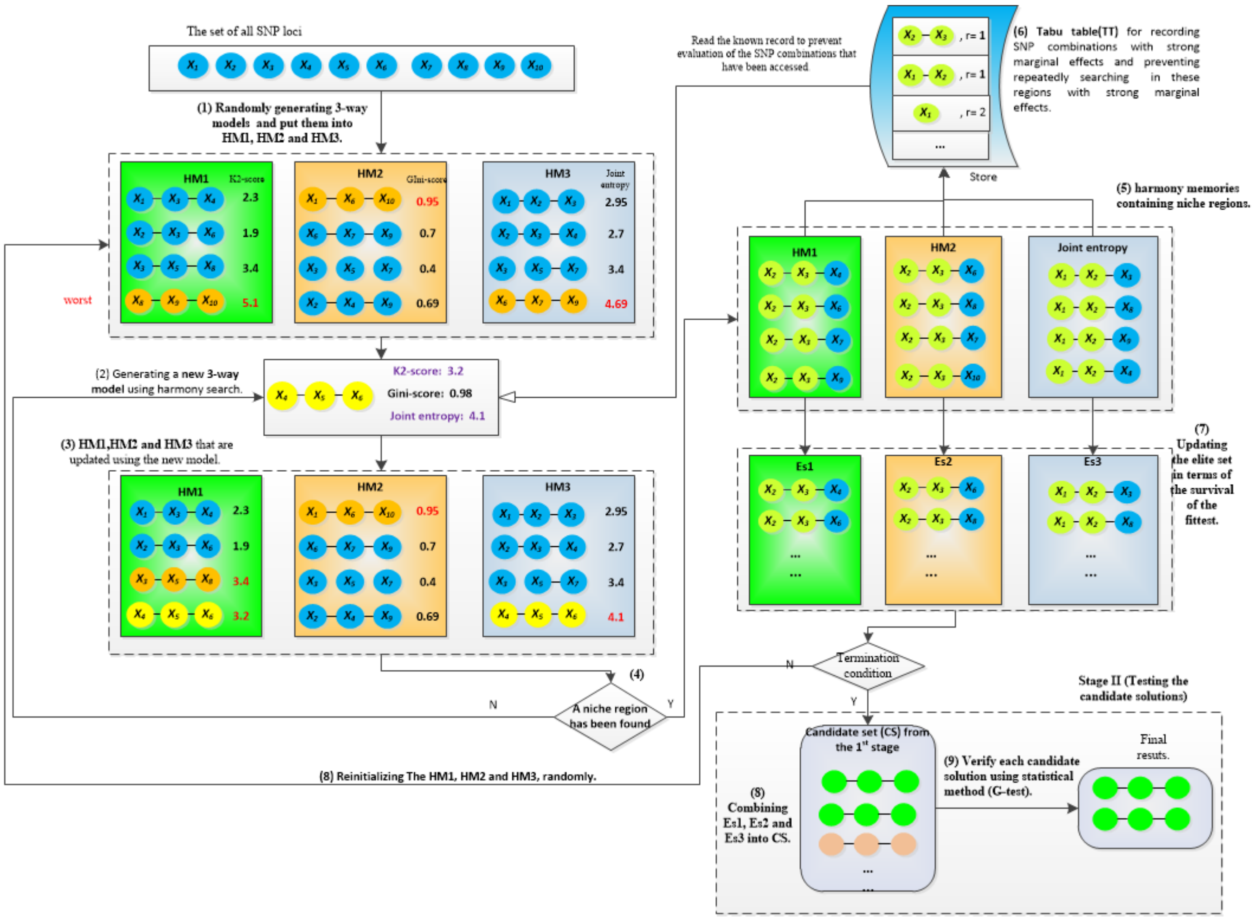

Fig A-5. Flowchart of steps involved in implementation of the NHSA-DHSC algorithm:

Fig A-5 presents an example explaining the process of the NHSA-DHSC algorithm for detecting 3-way SNP combination model with a total SNP number of 10. The process is divided into two stage, the first stage is for search candidate solutions, the second is for test the authenticity of each candidate solution.

Screening Stage: step (1)~step (8).

(1) There harmony memories (HM1, HM2 and HM3) are initialized randomly by selecting 3-way SNP combination from the set of all SNP loci, where each 3-way SNP combination is referred to as a harmony. Each harmony of HM1 is evaluated using K2-score, the harmony in HM2 is scored by Gini function and the method of joint entropy is used to evaluate the harmony of HM3.

(2) Generating a new 3-way model using harmony search algorithm and calculating the k2-score, Gini-score and joint entropy. Such as (X_4, X_5, X_6) (K2-score =3.2, Gini-score =0.98, Joint entropy=4.1).

(3) If the new generated 3-way model is superior to the worst harmony (with high K2-score, yellow color) in HM1, the worst harmony in HM1 is replaced by the new model. If the new model is better than the worst harmony in HM2 or HM3, it will replace the worst harmony. For example, in the HM1, the worst harmony is (X_8, X_9, X_{10}) whose K2-score=5.1, it is worse than (X_4, X_5, X_6), therefore, it will be replaced by the new harmony (X_4, X_5, X_6). Similarly, because harmony(X_4, X_5, X_6) is better than the worst harmony (X_6, X_7, X_9) of HM3, it will instead of the worst harmony in HM3. However, in HM2, the worst harmony (X_1, X_6, X_{10}) is better than new generated harmony(X_4, X_5, X_6), thus the HM2 cannot be updated.

(4) If the HM1, HM2 or HM3 have not contained many remarkably similar harmonies, go to step (2), otherwise, go to step (5).

(5) Finding new niche region according to the niche identification algorithm. For example (x_2, x_3) in HM1 and HM2, and (x_1, x_2) in HM3 are center of niche region, and the radius of them are equals to 1.

(6) Put the new identified niche into tabu table (TT) for avoiding the niche regions are searched repeatedly in later.

(7) Updating the elite set of candidate solutions. Select some best harmonies from HM1, HM2 and HM3, and update the elite set of candidate solutions (Es1, Es2, and Es3) in terms of mechanism for selecting the superior and eliminating the inferior.

(8) If termination condition is not satisfied, reinitializing the HM1, HM2 and HM3 randomly for finding new solutions, and the niche regions contained in TT are not searched in the future, otherwise, go to stage II.

Testing Stage: step (9)~step(10)

(9) Combining three elite sets (Es1, Es2, and Es3) into candidate set (CS) and removing the duplicate solutions.

(10) Testing the solutions using G-test statistical method.

4 Experimental data and results

Table E-1. The parameters and the values of penetrance of 12 DME models [12-13]

DME	H^2	MAF	D	AABB	AABb	AAbb	AaBB	AaBb	Aabb	aaBB	aaBb	aabb
DME -1	0.005	0.05	0.1	0.098	0.098	0.098	0.098	0.299	0.522	0.098	0.522	0.912
DME -2	0.005	0.1	0.1	0.096	0.096	0.096	0.096	0.197	0.282	0.096	0.282	0.405
DME -3	0.005	0.2	0.1	0.092	0.092	0.092	0.092	0.144	0.181	0.092	0.181	0.227
DME -4	0.005	0.5	0.1	0.078	0.078	0.078	0.078	0.105	0.122	0.078	0.122	0.142
DME -5	0.02	0.05	0.1	0.096	0.096	0.096	0.096	0.533	0.533	0.096	0.533	0.533
DME -6	0.02	0.1	0.1	0.092	0.092	0.092	0.092	0.319	0.319	0.092	0.319	0.319
DME -7	0.02	0.2	0.1	0.084	0.084	0.084	0.084	0.21	0.21	0.084	0.21	0.21
DME -8	0.02	0.5	0.1	0.052	0.052	0.052	0.052	0.137	0.137	0.052	0.137	0.137
DME -9	0.02	0.05	0.1	0.08	0.192	0.192	0.192	0.08	0.08	0.192	0.08	0.08
DME -10	0.02	0.1	0.1	0.072	0.164	0.164	0.164	0.072	0.072	0.164	0.072	0.072
DME -11	0.02	0.2	0.1	0.061	0.146	0.146	0.146	0.061	0.061	0.146	0.061	0.061
DME -12	0.02	0.5	0.1	0.067	0.155	0.155	0.155	0.067	0.067	0.155	0.067	0.067

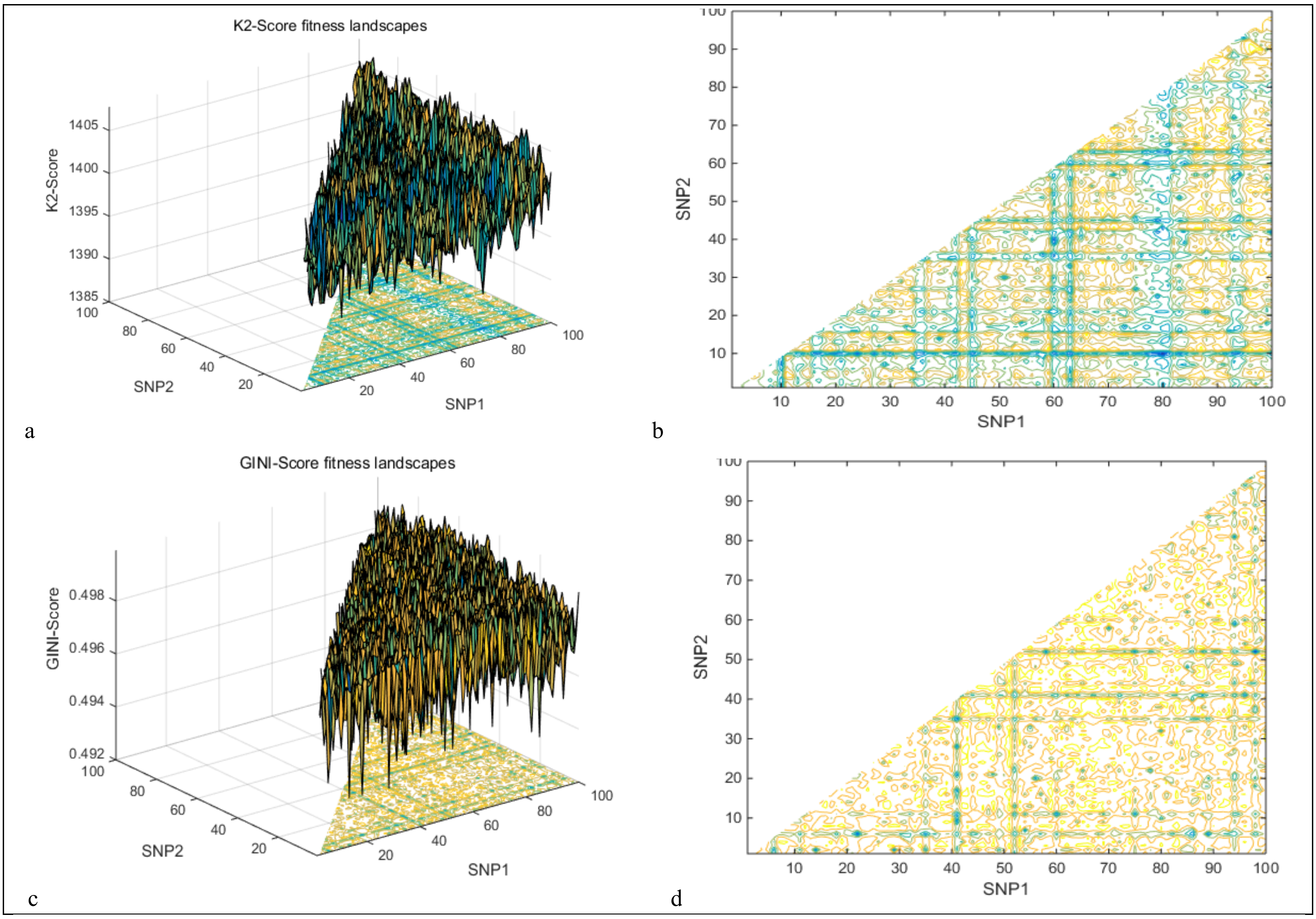

Fig.E-1. The fitness landscape of DME 1(H^2=0.005, MAF=0. 05). (a) Fitness curve surface based on K2-scoring criterion. (b) Fitness contour based on K2-scoring criterion. (c) Fitness curve surface based on GINI-scoring criterion. (d) Fitness contour based on GINI-scoring criterion. In the left curve figure, the lower the curve surface, the stronger association with phenotype the genotype combination (SNP1, SNP2) has; in the right contour figure, the deeper the color, the stronger association with phenotype the genotype combination (SNP1, SNP2) has.

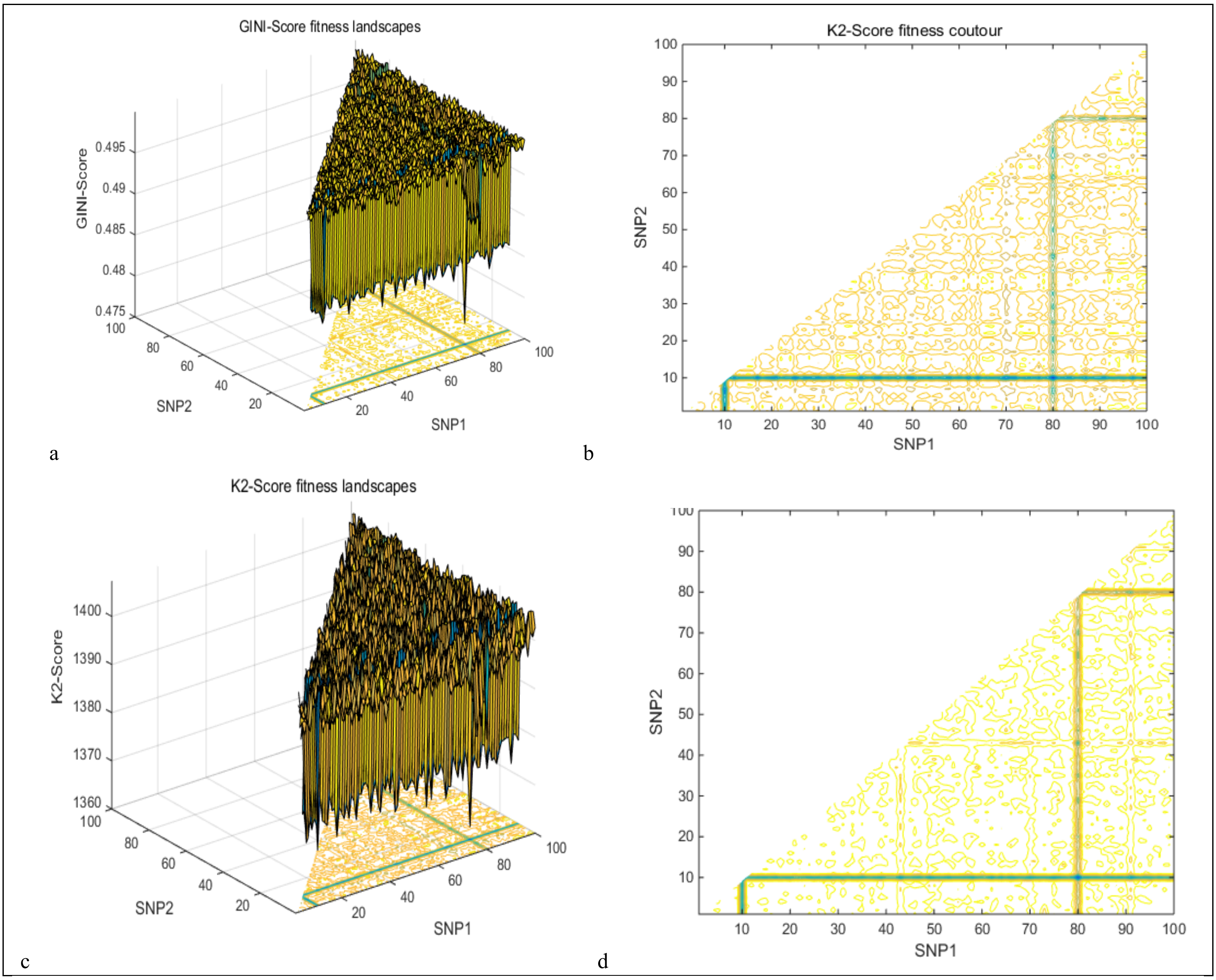

Fig.E-2. the fitness landscape of DME 8(H^2=0.02, MAF=0. 5). (a) fitness curve surface based on K2-scoring criterion. (b) Fitness contour based on K2-scoring criterion. (c) Fitness curve surface based on GINI-scoring criterion. (d) Fitness contour based on GINI-scoring criterion. In the right contour figure, the deeper the color, the stronger association with phenotype the genotype combination of (SNP1, SNP2) has. In the left curve figure, the lower the curve surface, the stronger association with phenotype the genotype combination (SNP1, SNP2) has; in the right contour figure, the deeper the color, the stronger association with phenotype the genotype combination (SNP1, SNP2) has.

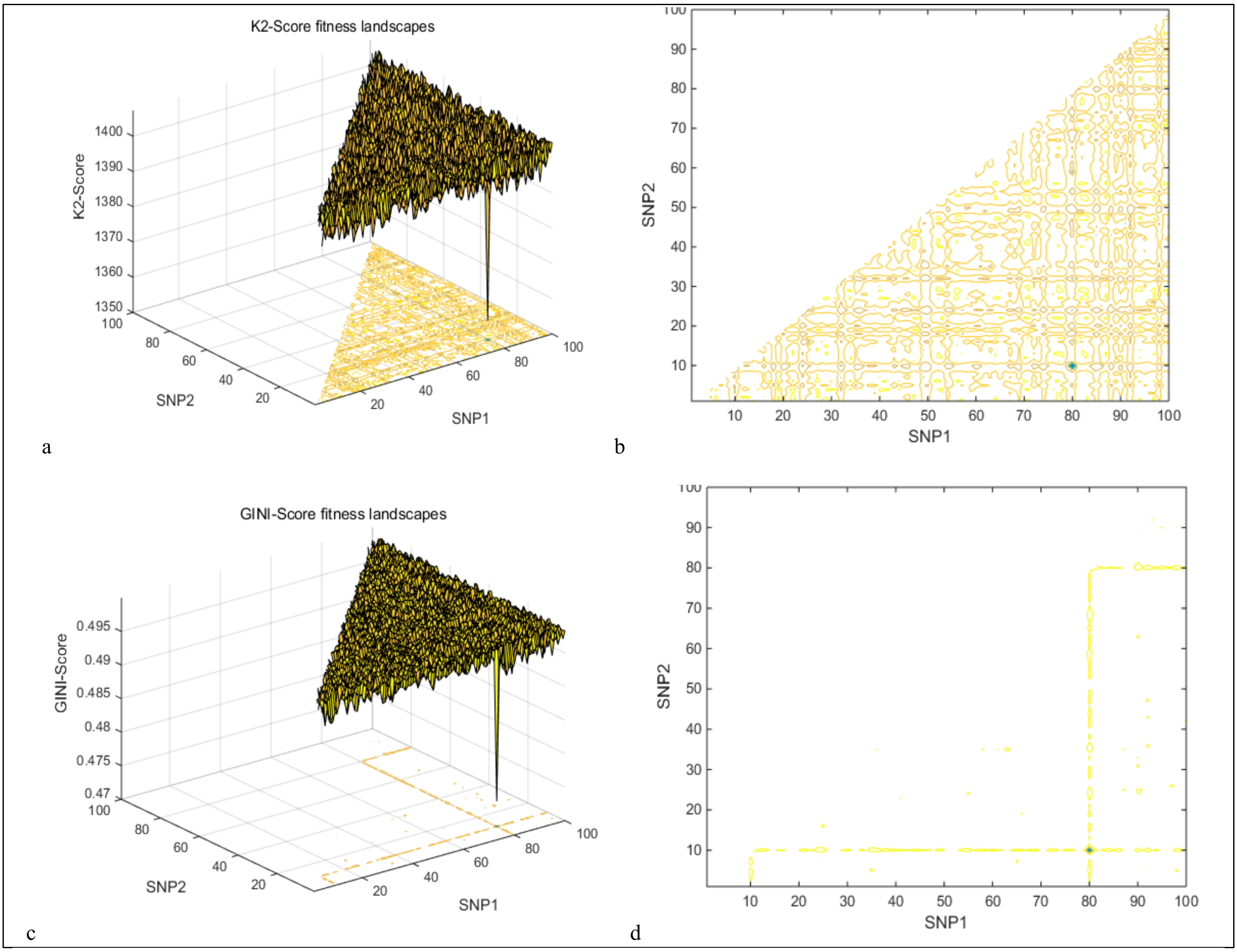

Fig.E-3. The fitness landscape of DME 12(H^2=0.02, MAF=0. 5). (a) Fitness curve surface based on K2-scoring criterion. (b) Fitness contour based on K2-scoring criterion. (c) Fitness curve surface based on GINI-scoring criterion. (d) Fitness contour based on GINI-scoring criterion. In the left curve figure, the lower the curve surface, the stronger association with phenotype the genotype combination (SNP1, SNP2) has; in the right contour figure, the deeper the color, the stronger association with phenotype the genotype combination (SNP1, SNP2) has.

Table E-2 the DNME models [14~15]

Model	k-way	HWE	One-way(sd)	Two-Way(sd)	Three-Way(sd)	Four-Way(sd)	Five-Way(sd)	tar.gz link
DNME-1	3	No	.502(.001)	.511(.007)	.886(.023)			threewayBests
DNME-2	3	Yes	.504(.002)	.509(.003)	.680(.024)			HWthreewayBests
DNME-3	4	No	.502(.001)	.510(.003)	-	.897(.018)		fourwayBests
DNME-4	4	Yes	.507(.003)	.513(.003)	-	.673(.009)		HWfourwayBests
DNME-5	4	No	.501(.000)	.504(.001)	.518(.003)	.567(.010)		fourwayNoLowBests
DNME-6	5	No	.502(.001)	.510(.002)	-	-	.895(.009)	fivewayBests
DNME-7	5	Yes	.511(.003)	.518(.003)	-	-	.693(.008)	HWfivewayBests
DNME-8	5	No	.503(.001)	.508(.001)	.518(.002)	.543(.004)	.690(.008)	fivewayNoLowBests

(1) Experimental results on Dataset with 100 SNP markers.

Table E-3 TPR, SPC, ACC and FDR of six algorithms for detecting datasets with 100SNPs .

In Table E-3 (see supplementary info file 2), some FDR values on the columns of SNPHarvester, Boost and MACOED equal “NaN”, which means that no k-way SNP combinations in all datasets were found to be a disease-causing model (Both the FP and TP are equal to 0, FP/(FP+TP) cannot be calculated) .

		NHSA-DHSC				MACOED				CSE				SNPHarvester				Beam				Boost			
**Sample size**	**Model**	**TPR**	**SPC**	**ACC**	**FDR**	**TPR**	**SPC**	**FDR**	**ACC**	**TPR**	**SPC**	**ACC**	**FDR**	**TPR**	**SPC**	**ACC**	**FDR**	**TPR**	**SPC**	**ACC**	**FDR**	**TPR**	**SPC**	**ACC**	**FDR**
800	DME-1	1%	100%	84%	0%	6%	100%	0%	68%	24%	24%	24%	76%	0%	100%	50%	**NaN**	0%	75%	43%	100%	0%	100%	50%	**NaN**
	DME-2	1%	100%	93%	75%	34%	100%	0%	78%	23%	23%	23%	77%	0%	100%	50%	**NaN**	0%	61%	38%	100%	0%	87%	44%	100%
	DME-3	1%	100%	95%	67%	73%	98%	7%	90%	11%	11%	11%	89%	0%	100%	50%	**NaN**	0%	68%	40%	100%	0%	100%	50%	**NaN**
	DME-4	2%	100%	95%	82%	94%	80%	30%	85%	6%	6%	6%	94%	0%	100%	50%	**NaN**	6%	46%	33%	95%	0%	100%	50%	**NaN**
	DME-5	24%	100%	77%	0%	98%	95%	6%	96%	63%	63%	63%	37%	0%	100%	50%	**NaN**	23%	56%	42%	73%	5%	100%	53%	0%
	DME-6	31%	99%	91%	14%	100%	98%	2%	99%	68%	68%	68%	32%	0%	100%	50%	**NaN**	26%	42%	36%	80%	1%	100%	51%	0%
	DME-7	63%	98%	91%	13%	99%	81%	19%	89%	66%	66%	66%	34%	1%	98%	50%	67%	79%	10%	34%	69%	2%	98%	50%	50%
	DME-8	93%	97%	95%	2%	100%	71%	29%	83%	62%	62%	62%	38%	31%	89%	61%	28%	100%	0%	69%	31%	0%	98%	49%	100%
	DME-9	76%	100%	89%	0%	99%	79%	21%	88%	82%	82%	82%	18%	4%	100%	52%	0%	88%	12%	50%	50%	0%	98%	49%	100%
	DME-10	90%	100%	95%	0%	99%	73%	28%	84%	77%	77%	77%	23%	13%	100%	57%	0%	87%	22%	63%	35%	13%	100%	57%	0%
	DME-11	40%	100%	97%	2%	100%	100%	0%	100%	76%	76%	76%	24%	15%	100%	58%	0%	11%	70%	44%	78%	87%	100%	94%	0%
	DME-12	52%	100%	96%	6%	99%	71%	29%	83%	75%	75%	75%	25%	19%	100%	60%	0%	19%	35%	30%	89%	30%	98%	64%	6%
1600	DME-1	4%	100%	82%	0%	15%	99%	8%	70%	54%	54%	54%	46%	0%	100%	50%	**NaN**	0%	79%	44%	100%	0%	100%	50%	**NaN**
	DME-2	3%	100%	93%	57%	68%	99%	3%	87%	46%	46%	46%	54%	0%	100%	50%	**NaN**	3%	68%	41%	94%	2%	100%	51%	0%
	DME-3	15%	100%	94%	30%	93%	99%	2%	97%	38%	38%	38%	62%	0%	100%	50%	**NaN**	10%	56%	38%	88%	0%	100%	50%	**NaN**
	DME-4	27%	99%	93%	26%	98%	79%	24%	87%	25%	25%	25%	75%	1%	99%	50%	50%	46%	26%	33%	77%	0%	100%	50%	**NaN**
	DME-5	79%	100%	91%	0%	96%	99%	1%	98%	78%	78%	78%	22%	0%	100%	50%	**NaN**	64%	42%	54%	44%	25%	100%	63%	0%
	DME-6	92%	100%	98%	0%	98%	87%	13%	92%	74%	74%	74%	26%	34%	97%	66%	8%	79%	28%	57%	41%	61%	99%	80%	2%
	DME-7	97%	98%	98%	2%	98%	82%	18%	89%	83%	83%	83%	17%	73%	41%	51%	64%	98%	6%	72%	27%	49%	99%	74%	2%
	DME-8	100%	100%	100%	0%	100%	64%	36%	78%	75%	75%	75%	25%	100%	2%	12%	90%	97%	15%	83%	15%	4%	100%	52%	0%
	DME-9	99%	100%	99%	0%	99%	74%	26%	85%	80%	80%	80%	20%	77%	29%	40%	74%	100%	0%	83%	17%	0%	100%	50%	**NaN**
	DME-10	99%	100%	99%	0%	100%	83%	17%	91%	81%	81%	81%	19%	94%	47%	63%	53%	100%	0%	83%	17%	67%	100%	84%	0%
	DME-11	68%	100%	97%	1%	99%	97%	4%	98%	78%	78%	78%	22%	96%	100%	98%	0%	27%	45%	38%	76%	100%	100%	100%	0%
	DME-12	91%	99%	97%	4%	97%	86%	14%	91%	79%	79%	79%	21%	98%	55%	71%	44%	98%	2%	58%	41%	98%	99%	99%	1%
2000	DME-1	12%	100%	79%	0%	0%	100%	**NaN**	84%	60%	60%	60%	40%	0%	100%	50%	**NaN**	8%	76%	45%	79%	0%	97%	49%	100%
	DME-2	13%	100%	93%	13%	4%	99%	33%	78%	55%	55%	55%	45%	0%	100%	50%	**NaN**	2%	55%	36%	98%	12%	98%	55%	14%
	DME-3	22%	100%	94%	15%	35%	99%	13%	90%	44%	44%	44%	56%	1%	100%	51%	0%	13%	41%	32%	90%	0%	100%	50%	**NaN**
	DME-4	45%	99%	94%	10%	60%	92%	52%	88%	28%	28%	28%	72%	0%	100%	50%	**NaN**	70%	17%	37%	67%	0%	100%	50%	**NaN**
	DME-5	90%	100%	95%	0%	61%	100%	0%	84%	74%	74%	74%	26%	0%	100%	50%	**NaN**	71%	44%	61%	34%	66%	98%	82%	3%
	DME-6	95%	100%	98%	0%	93%	93%	9%	93%	74%	74%	74%	26%	58%	84%	72%	25%	91%	19%	69%	28%	65%	100%	83%	0%
	DME-7	99%	86%	96%	4%	100%	84%	16%	91%	73%	73%	73%	27%	95%	16%	30%	80%	100%	0%	78%	22%	66%	98%	82%	3%
	DME-8	100%	100%	100%	0%	97%	86%	14%	91%	70%	70%	70%	30%	100%	1%	15%	85%	99%	6%	85%	14%	9%	100%	55%	0%
	DME-9	100%	100%	100%	0%	99%	85%	15%	92%	77%	77%	77%	23%	98%	6%	13%	92%	99%	8%	88%	11%	8%	88%	48%	60%
	DME-10	99%	100%	99%	0%	97%	85%	16%	90%	82%	82%	82%	18%	99%	18%	32%	79%	98%	13%	87%	12%	87%	98%	93%	2%
	DME-11	82%	100%	98%	2%	98%	84%	16%	90%	66%	66%	66%	34%	99%	100%	100%	0%	55%	32%	42%	63%	99%	100%	100%	0%
	DME-12	98%	100%	99%	0%	97%	81%	20%	88%	73%	73%	73%	27%	100%	41%	59%	57%	91%	16%	64%	35%	100%	100%	100%	0%

		**NHSA-DHSC**				**MACOED**				**CSE**				**SNPHarvester**				**Beam**				**Boost**			
**Sample size**	**Model**	**TPR**	**SPC**	**ACC**	**FDR**	**TPR**	**SPC**	**FDR**	**ACC**	**TPR**	**SPC**	**ACC**	**FDR**	**TPR**	**SPC**	**ACC**	**FDR**	**TPR**	**SPC**	**ACC**	**FDR**	**TPR**	**SPC**	**ACC**	**FDR**
3200	DME-1	19%	100%	81%	0%	0%	100%	NaN	93%	75%	75%	75%	25%	0%	100%	50%	**NaN**	5%	73%	44%	87%	4%	96%	50%	50%
	DME-2	48%	100%	95%	0%	0%	100%	NaN	93%	69%	69%	69%	31%	2%	100%	51%	0%	27%	47%	39%	75%	11%	100%	56%	0%
	DME-3	70%	99%	96%	6%	8%	100%	0%	96%	65%	65%	65%	35%	8%	99%	54%	11%	60%	26%	39%	66%	8%	95%	52%	38%
	DME-4	85%	100%	96%	0%	6%	100%	50%	95%	63%	63%	63%	37%	25%	58%	46%	73%	97%	5%	63%	36%	0%	100%	50%	**NaN**
	DME-5	99%	100%	99%	0%	0%	99%	100%	75%	76%	76%	76%	24%	65%	100%	83%	0%	90%	26%	72%	24%	94%	97%	96%	3%
	DME-6	99%	100%	99%	0%	7%	100%	0%	72%	78%	78%	78%	22%	100%	36%	55%	61%	99%	6%	84%	16%	99%	100%	100%	0%
	DME-7	100%	100%	100%	0%	60%	93%	27%	85%	67%	67%	67%	33%	100%	4%	19%	83%	95%	31%	86%	10%	96%	96%	96%	4%
	DME-8	100%	100%	100%	0%	83%	74%	37%	77%	77%	77%	77%	23%	100%	0%	28%	72%	97%	19%	86%	12%	37%	94%	66%	14%
	DME-9	100%	100%	100%	0%	39%	99%	3%	74%	79%	79%	79%	21%	100%	2%	46%	55%	97%	20%	87%	11%	32%	100%	66%	0%
	DME-10	100%	100%	100%	0%	70%	99%	1%	86%	82%	82%	82%	18%	100%	2%	13%	89%	95%	24%	83%	14%	99%	100%	100%	0%
	DME-11	93%	99%	98%	3%	83%	100%	0%	93%	81%	81%	81%	19%	99%	88%	93%	12%	75%	29%	54%	44%	95%	97%	96%	3%
	DME-12	100%	100%	100%	0%	81%	99%	2%	94%	76%	76%	76%	24%	100%	4%	10%	94%	96%	17%	80%	18%	88%	88%	88%	12%
4000	DME-1	50%	99%	84%	6%	0%	100%	NaN	88%	78%	78%	78%	22%	0%	100%	50%	**NaN**	17%	60%	42%	77%	24%	94%	59%	20%
	DME-2	78%	100%	96%	1%	0%	100%	NaN	87%	78%	78%	78%	22%	9%	100%	55%	0%	52%	45%	49%	53%	46%	94%	70%	12%
	DME-3	82%	100%	97%	1%	11%	98%	50%	88%	74%	74%	74%	26%	38%	95%	67%	12%	93%	7%	50%	50%	35%	98%	67%	5%
	DME-4	95%	100%	98%	0%	57%	98%	35%	95%	63%	63%	63%	37%	52%	28%	34%	81%	100%	0%	75%	25%	0%	100%	50%	**NaN**
	DME-5	98%	100%	98%	0%	27%	100%	0%	70%	86%	86%	86%	14%	96%	100%	98%	0%	100%	0%	88%	12%	98%	99%	99%	1%
	DME-6	100%	100%	100%	0%	80%	99%	1%	90%	78%	78%	78%	22%	100%	8%	18%	88%	100%	0%	85%	15%	98%	98%	98%	2%
	DME-7	100%	100%	100%	0%	99%	86%	17%	91%	80%	80%	80%	20%	100%	0%	10%	90%	100%	0%	93%	7%	100%	100%	100%	0%
	DME-8	100%	100%	100%	0%	97%	67%	33%	79%	78%	78%	78%	22%	100%	0%	15%	85%	100%	0%	92%	8%	57%	100%	79%	0%
	DME-9	100%	100%	100%	0%	94%	91%	10%	92%	82%	82%	82%	18%	100%	0%	16%	84%	100%	0%	90%	10%	48%	99%	73%	2%
	DME-10	100%	100%	100%	0%	96%	82%	21%	87%	77%	77%	77%	23%	100%	2%	25%	76%	100%	0%	91%	9%	98%	98%	98%	2%
	DME-11	98%	100%	99%	0%	100%	100%	0%	100%	81%	81%	81%	19%	100%	100%	100%	0%	92%	15%	65%	33%	100%	100%	100%	0%
	DME-12	100%	100%	100%	0%	99%	97%	4%	98%	78%	78%	78%	22%	100%	3%	15%	87%	100%	0%	88%	12%	99%	100%	100%	0%
5000	DME-1	69%	100%	87%	0%	46%	100%	0%	77%	75%	75%	75%	25%	2%	100%	51%	0%	36%	65%	50%	49%	57%	100%	79%	0%
	DME-2	92%	100%	98%	0%	84%	99%	3%	93%	75%	75%	75%	25%	27%	100%	64%	0%	56%	36%	45%	58%	52%	92%	72%	14%
	DME-3	92%	100%	98%	0%	99%	95%	6%	97%	76%	76%	76%	24%	57%	98%	78%	3%	86%	20%	58%	41%	24%	100%	62%	0%
	DME-4	100%	100%	100%	0%	100%	89%	12%	94%	71%	71%	71%	29%	84%	7%	15%	91%	97%	17%	84%	14%	0%	99%	50%	100%
	DME-5	97%	100%	98%	0%	98%	86%	16%	91%	74%	74%	74%	26%	99%	88%	93%	12%	99%	6%	86%	14%	98%	98%	98%	2%
	DME-6	100%	90%	96%	7%	100%	83%	17%	91%	76%	76%	76%	24%	100%	8%	27%	78%	91%	53%	85%	8%	99%	99%	99%	1%
	DME-7	100%	100%	100%	0%	99%	67%	33%	80%	81%	81%	81%	19%	100%	0%	44%	56%	98%	18%	90%	8%	94%	94%	94%	6%
	DME-8	100%	100%	100%	0%	99%	68%	32%	81%	82%	82%	82%	18%	100%	0%	14%	86%	98%	13%	87%	12%	58%	100%	79%	0%
	DME-9	100%	100%	100%	0%	97%	76%	24%	85%	81%	81%	81%	19%	100%	0%	35%	65%	99%	10%	91%	8%	45%	100%	73%	0%
	DME-10	100%	100%	100%	0%	99%	78%	22%	87%	75%	75%	75%	25%	100%	0%	12%	88%	92%	47%	85%	9%	100%	100%	100%	0%
	DME-11	97%	100%	99%	1%	98%	89%	13%	93%	78%	78%	78%	22%	100%	84%	92%	15%	99%	3%	71%	29%	100%	100%	100%	0%
	DME-12	99%	90%	94%	9%	100%	76%	24%	87%	75%	75%	75%	25%	100%	0%	12%	88%	100%	0%	93%	7%	96%	96%	96%	4%

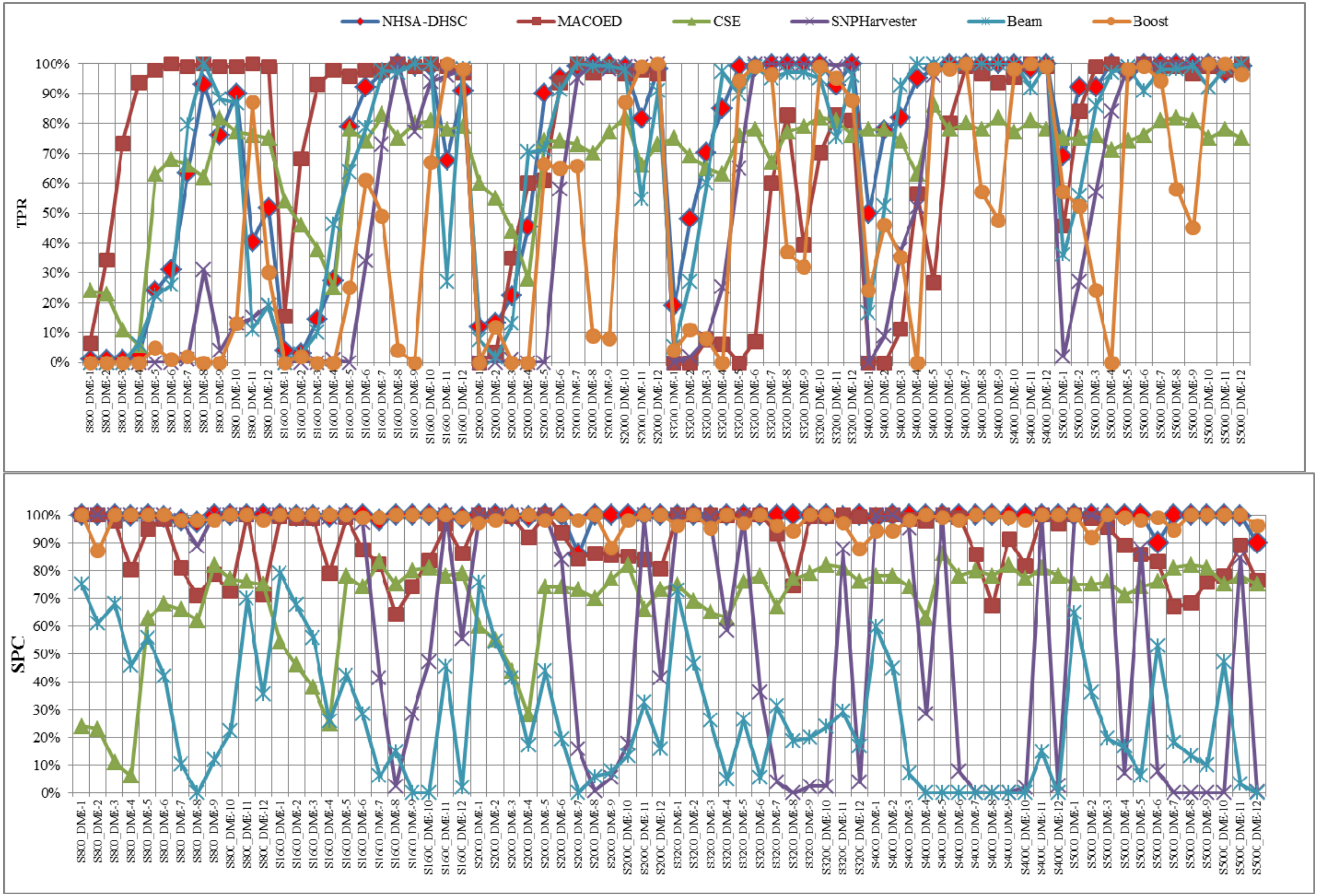

Figure E-4. SPC and TPR of six algorithms for detecting datasets with 100SNPs and 4000 samples.

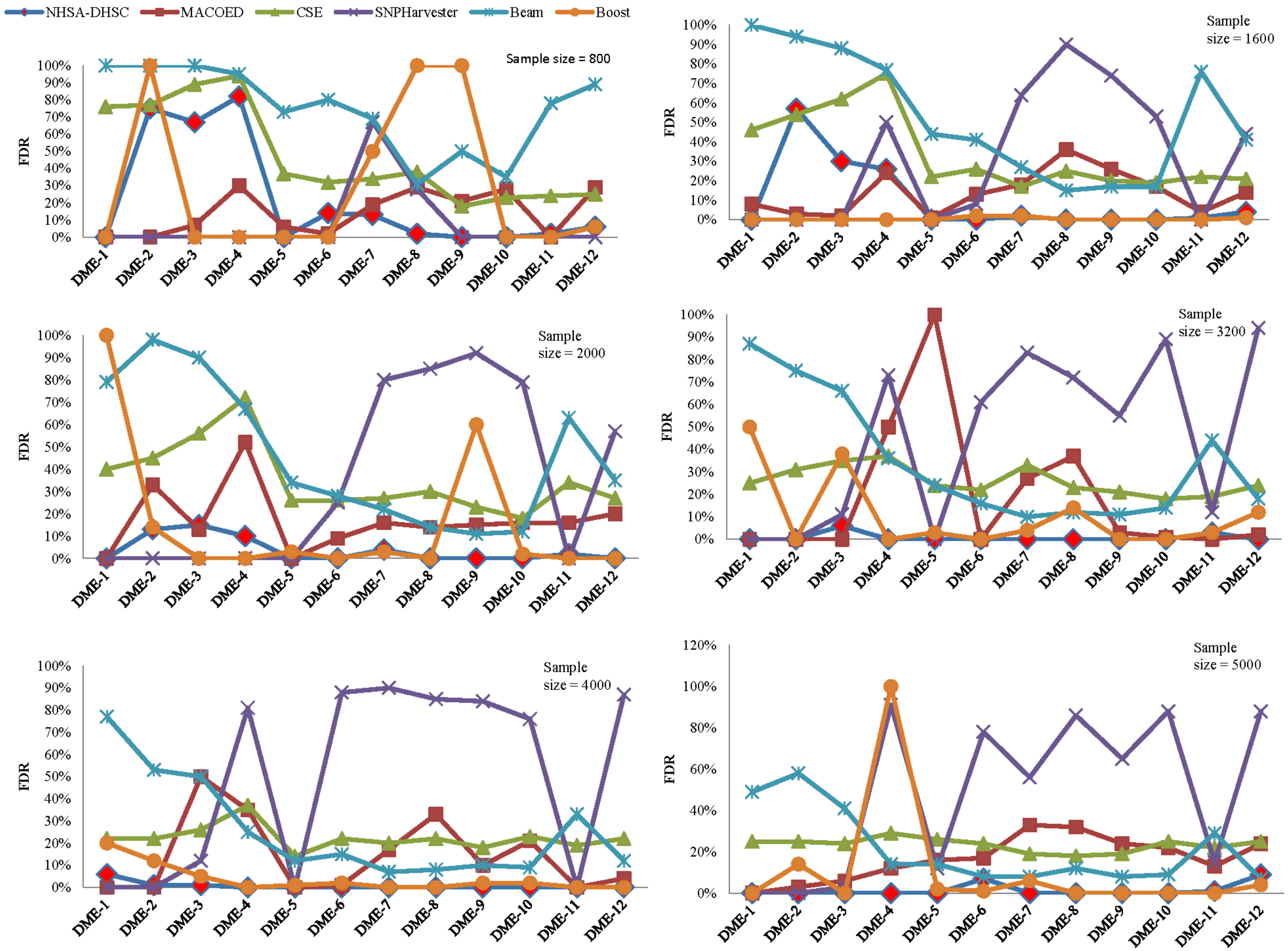

Figure E-5. Comparison FDR of six algorithms on datasets with 100SNPs.

(2) Experimental results on datasets with 1000 SNP markers.

We further investigate the detection power of our method for datasets with 1000 SNP markers and 4000 samples. The comparison of six algorithms on powers, number of evaluations (Es) and runtime are shown in **Figure E-6**. **Figure E-7** presents four statistical metrics ACC, TPR, SPC and FDR of six algorithms. In addition, we investigate the performance of G-test employed in our method by comparing with chi-square test, where the chi-square also tests the authenticity of the candidate solutions screened from first stage using our method. **Figure E-8** shows the TPR, SPC, ACC and FDR of G-test and chi-square using identical threshold p-value.

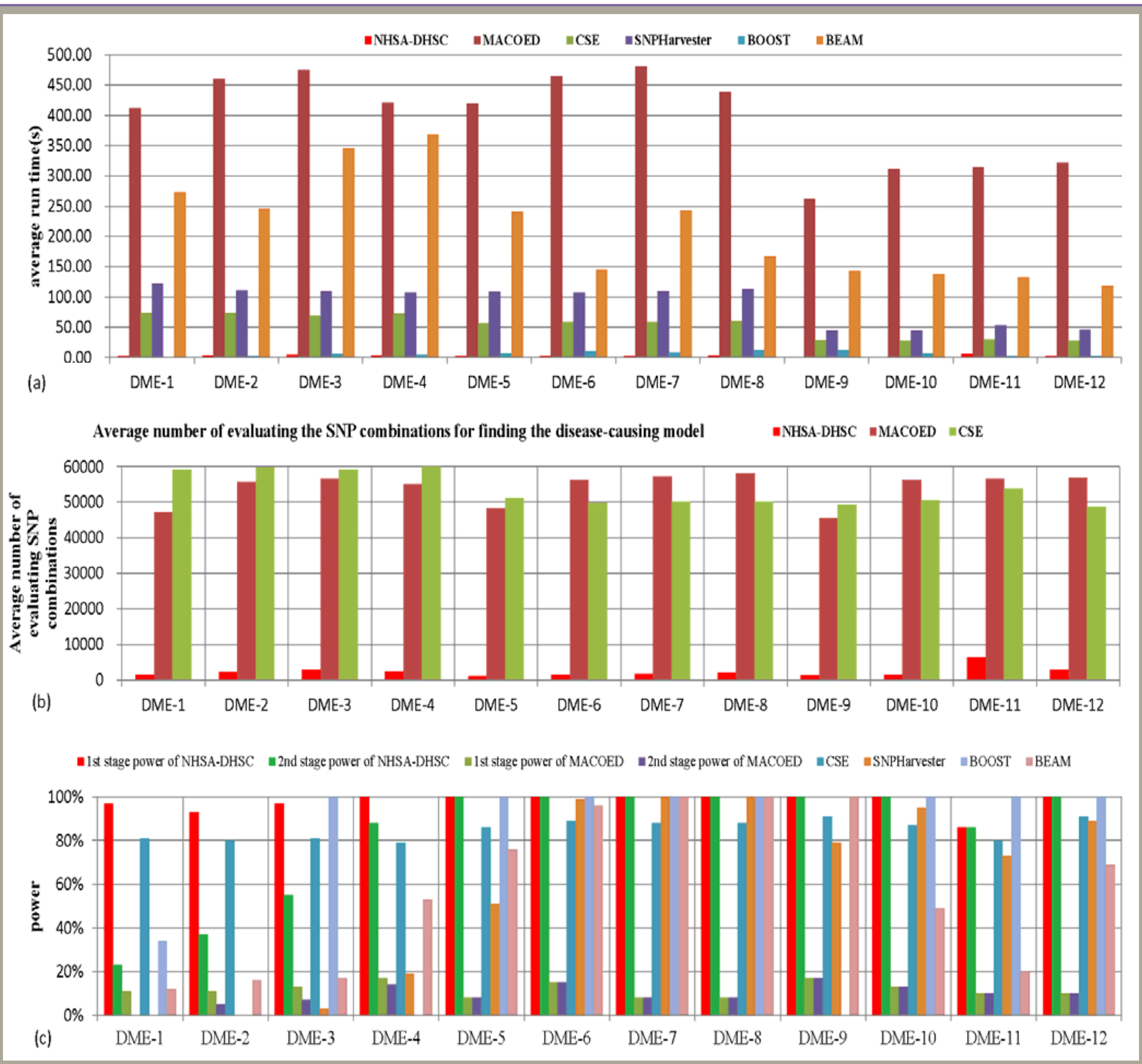

Figure E-6. Comparison on Power, Runtime and number of Evaluations for find the disease-causing models from dataset with 1000SNPs and 4000 samples.

Figure (a) shows the average run time for finding the disease-causing models.

Figure (b) shows the average number of SNP combinations evaluated by the three intelligent search algorithms to detect disease models.

Figure (c) displays the power of six algorithms for detecting 12 DME models, where NHSA-DHSC and MACOED contain two stages.

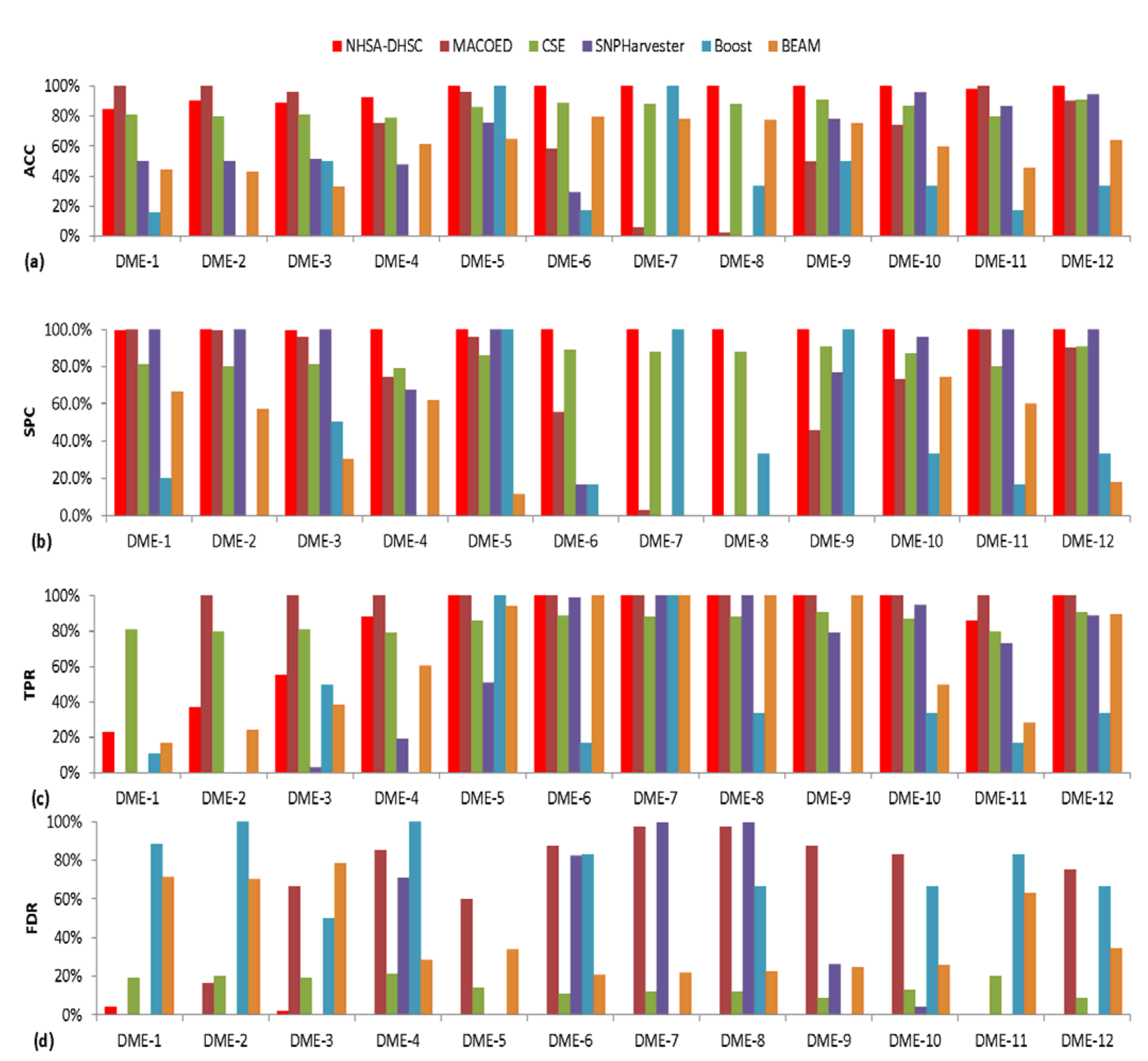

Figure E-7. Comparison of six algorithms on ACC, SPC, TPR, FDR for detecting datasets with 1000SNPs and 4000 samples.

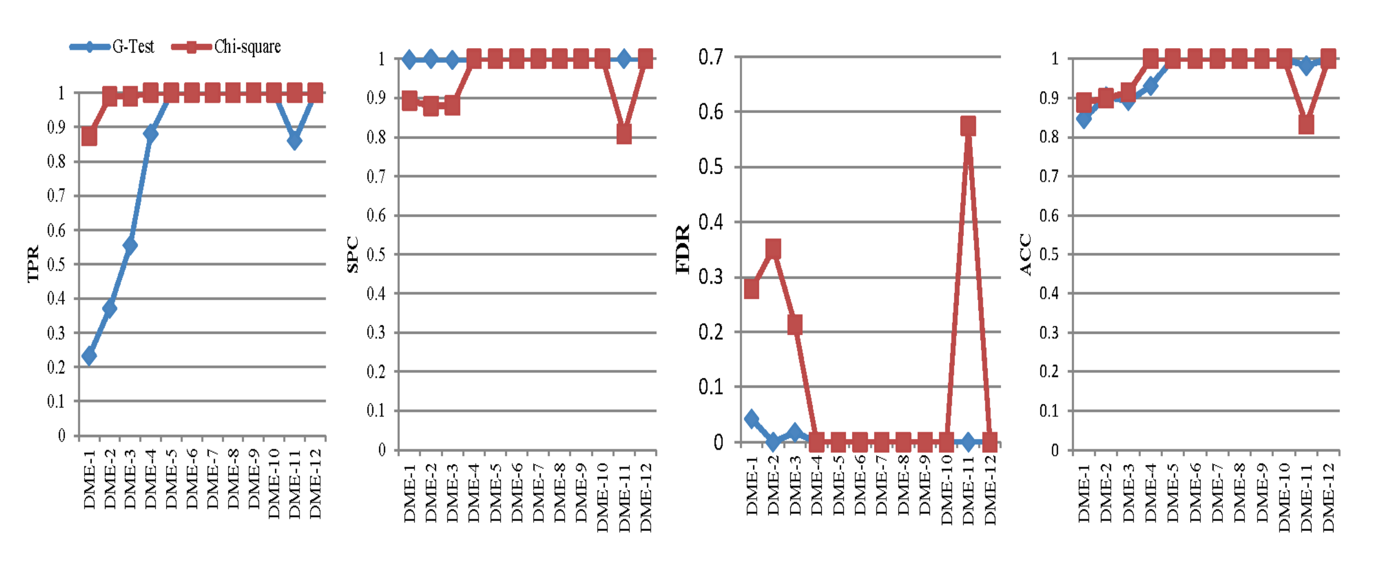

Figure E-8. The statistical precision comparison between G-test and chi-square test.
In this figure, we respectively employ G-test and chi-square test to verify the solutions of candidate set (CS) in the 1st stage of NHSA-DHSC, where 12 DME datasets with 1000 SNP markers and 4000 samples are tested.

For datasets with 1000 SNP markers, as shown in Figure E-6, our method in the first stage possesses the highest detection power and takes least runtime for all 12 DME models. In Figure E-6(c), NHSA-DHSC uses the least number of MEs to obtain the disease-causing SNP combinations among three intelligent algorithms.

It can be seen from Figure E-7(a) that our method is superior to other five algorithms for DME-4~DME-12 on index ACC, and it is better than CSE, Beam, SNPHarvester and Boost on DME-1~DME-3. In Figure E-7(b), our method is superior to or equal to all other approaches on the SPC metric, and it shows very high true negative rate in Figure E-7(c) for DME-5~DME-12, but it is inferior to MACOED and CSE for DME-1~DME-4, which is because many true disease models obtained in the first stage are filtered out due to the failure to pass the threshold of G-test p-value. In **Figure E-7**(d), the NHSA-DHSC shows very low FDR, which demonstrates our method is effective for decreasing the type I error.

We can see from **Figure E-8** that the G-test has higher precision on SPC and FDR than chi-square test. For DME-1~DME-4, the TPR of G-test is inferior to that of chi-square test, which is because the G-test is more stringent than chi-square. As we known, statistical method must balance the increase in type I errors and the increase in type II errors. A stringent significant threshold can decrease the type I errors for some models, however, which also can decrease the power (type II errors) for other models, such as DME models 1-4.Therefore, we believe that the modified G-test in this work is more effective than chi-square for testing the candidate solutions.

Reference

[1]. Z.W. Geem, J. Kim, G. Loganathan, Music-inspired optimization algorithm harmony search, *Simulation* **76**, 60-68(2001).

[2]. Zong, W. G. Novel derivative of harmony search algorithm for discrete design variables. Applied Mathematics & Computation 199(1), 223-230(2008).

[3]. E.L. Yu, P.N. Suganthan. Ensemble of niching algorithms. information sciences 180, 2815–2833(2010).

[4]. Mostafa Z. Ali, Noor H. Awad. A novel class of niche hybrid Cultural Algorithms for continuous engineering optimization. information sciences 267, 158–190 (2014).

[5]. Zhang, Y., & Liu, J. S. Bayesian inference of epistatic interactions in case-control studies （BEAM）. Nature genetics, 39(9), 1167-1173 (2007)..

[6]. Jiang, X., Neapolitan, R. E., Barmada, M. M., & Visweswaran, S. Learning genetic epistasis using Bayesian network scoring criteria. BMC bioinformatics 12(1), 89(2011).

[7]. Visweswaran S, Wong AI, Barmada MM. A Bayesian method for identifying genetic interactions. Proceedings of the Fall Symposium of the American Medical Informatics Association 2009, 673-677（2009）.

[8]. Cooper GF, Herskovits E. A bayesian method for the induction of probabilistic networks from data. Machine Learning 9(4), 309-347(1992).

[9]. Peng-Jie Jing and Hong-Bin Shen, MACOED: A multi-objective ant colony optimization algorithm for SNP epistasis detection in genome-wide association studies, Bioinformatics 31: 634-641(2015).

[10]. Himmelstein et al. Evolving hard problems: Generating human genetics datasets with a complex etiology. BioData Mining 4, 21(2011). doi:10.1186/1756-0381-4-21. http://discovery.dartmouth.edu/model_free_data/.

[11]. Breiman, L., Friedman, J., Olshen, R., & Stone, C. Classification and regression trees. belmont, ca: wadsworth international group. Biometrics, 40(3), 17–23(1984).

[12]. Jing, P. J., & Shen, H. B. Macoed: a multi-objective ant colony optimization algorithm for snp epistasis detection in genome-wide association studies. Bioinformatics 31(5), 634(2015).

[13]. Urbanowicz, R. J., Kiralis, J., Sinnottarmstrong, N. A., Heberling, T., Fisher, J. M., et al. Gametes: a fast, direct algorithm for generating pure, strict, epistatic models with random architectures. BioData Mining, 5(1), 16(2012).

[14]. Velez, D. R., White, B. C., Motsinger, A. A., Bush, W. S., Ritchie, M. D., Williams, S. M. et al. (2007), A balanced accuracy function for epistasis modeling in imbalanced datasets using multifactor dimensionality reduction. Genet. Epidemiol (supplymentary), 31: 306–315. doi: 10.1002/gepi.20211

[15]. Himmelstein, D. S., Greene, C. S., & Moore, J. H. Evolving hard problems: generating human genetics datasets with a complex etiology. BioData Mining, 4(1), 1-13 (2011). doi:10.1186/1756-0381-4-21. http://discovery.dartmouth.edu/model_free_data/.
